# Imaging biomarkers related to 18F-FDG PET/CT for predicting microvascular invasion in hepatocellular carcinoma: a systematic review and meta-analysis

**DOI:** 10.3389/fonc.2026.1948193

**Published:** 2026-10-12

**Authors:** Zhigang Li, Ke Gong, Ning Liu

**Affiliations:** 1 Department of General Surgery, Xiaogan Hospital Affiliated to Wuhan University of Science and Technology, Xiaogan, China; 2 Medical College, Wuhan University of Science and Technology, Wuhan, China

**Keywords:** 18F-FDG, biomarkers, hepatocellular carcinoma, microvascular invasion, PET/CT

## Abstract

**Introduction:**

This study, through a meta-analysis, aimed to assess the predictive performance of 18F-fluorodeoxyglucose positron emission tomography/computed tomography (18F-FDG PET/CT) for microvascular invasion (MVI) in hepatocellular carcinoma (HCC).

**Methods:**

Web of Science, Embase, PubMed, and Cochrane Library were comprehensively searched from database inception to January 13, 2026. EndNote 21was employed for literature screening and data extraction. Meta-analysis was carried out on the extracted data using Stata 15, MetaDisc 1.4, and MedCalc.

**Results:**

In total, 21 studies were included, involving 2,023 patients with HCC, of whom 890 were MVI+ and 1,133 were MVI–. In univariable analyses, both tumor-to-normal liver uptake ratio (TLR) (odds ratio [OR] = 2.577, 95% confidence interval [CI]: 1.446–4.593, P = 0.001) and maximum standardized uptake value (SUVmax) (OR = 1.272, 95% CI: 1.036–1.561, P = 0.021) were significantly associated with an increased MVI risk. After multivariable adjustment, SUVmax retained independent predictive value (OR = 2.140, 95% CI: 1.244–3.681, P = 0.006), whereas the multivariable evidence for TLR was derived from a single study and required further validation. AUC values for SUVmax and radiomics score (Rad-score) were 0.819 (95% CI: 0.739–0.899) and 0.809 (95% CI: 0.762–0.857), respectively, with relatively balanced values for sensitivity, specificity, and negative likelihood ratio.

**Conclusion:**

The available evidence supports SUVmax as one of the metabolic markers for preoperative MVI risk assessment in HCC, with Rad-score similar to SUVmax in diagnostic discrimination. The complementary value of the two markers was not demonstrated by this Meta-analysis, but rather derived from inferences proposed by external studies. Moreover, because the included radiomics studies did not report a pooled effect size for the combined model integrating SUVmax and Rad-score, a Meta-analysis targeting this model is currently not feasible. Therefore, the complementarity of SUVmax and Rad-score should be regarded as a hypothesis to be tested. In the future, multicenter, prospective radiomics studies with rigorous external validation are warranted to further explore a combined prediction model based on SUVmax and Rad-score. On this basis, an integrated assessment workflow should be gradually established, by which preoperative decisions are guided.

**Systematic Review Registration:**

https://www.crd.york.ac.uk/PROSPERO/, identifier CRD420261289724.

## 1 Introduction

Hepatocellular carcinoma (HCC) is a malignant tumor featuring a high incidence rate and a high mortality rate worldwide, accounting for over 500,000 deaths annually (1). Chronic hepatitis B or C is the primary etiology of HCC, contributing to 80% of all HCC cases globally (2). Microvascular invasion (MVI) is of vital importance among the pathological features of HCC. MVI features clusters of carcinoma cells located within vascular channels surrounded by endothelial cells, and these clusters are most typically found within the branches of the peripheral portal vein capsules. MVI is widely recognized as an independent risk factor for postoperative recurrence and poor prognosis (3). Nevertheless, MVI is primarily detected through immunohistochemistry and pathological analysis of tissue samples after surgery, which are invasive and limited in scope, and an effective non-invasive prediction method before surgery is lacking.

As a functional-anatomical fusion imaging technique, 18F-fluorodeoxyglucose positron emission tomography/computed tomography (18F-FDG PET/CT) operates on the principle that 18F-FDG is a glucose metabolism tracer whose biological behavior in the body resembles that of glucose. When injected into the body, 18F-FDG is transported into cells similarly to glucose and converted into 6-p-18FDG by hexokinase. 6-p-18FDG is not further metabolized but remains trapped within cells. Accordingly, the uptake of 18F-FDG by cells is proportional to the glucose metabolism rate, which reflects the glucose metabolism activity of tumors and is potentially effective in predicting vascular invasion (4). In recent years, studies have explored the value of PET/CT-based metabolic parameters, radiomic features, and the prediction models constructed from them for non-invasively predicting MVI status in HCC before surgery, exhibiting promising potential in application (5, 6). Nevertheless, existing studies are mostly retrospective investigations based on single-center data and with a small sample size, and discrepancies are observed in the prediction indicators, thresholds, and diagnostic performance adopted across studies. Accordingly, no unified conclusion has been reached.

Therefore, this study, through a systematic review and meta-analysis, aims to systematically and quantitatively assess the overall diagnostic performance of indicators related to 18F-FDG PET/CT for MVI among individuals with HCC. As a consequence, this study may provide higher-level evidence-based medical support for preoperative decision-making in clinical settings.

## 2 Methods

This study was conducted adhering to the Preferred Reporting Items for Systematic Reviews and Meta-Analyses (PRISMA) guidelines (**Appendix 1**), and the study protocol was registered with the International Prospective Register of Systematic Reviews (PROSPERO) in January 2026 (registration number: CRD420261289724).

### 2.1 Literature search

PubMed, Web of Science, Embase, and Cochrane Library were comprehensively searched to identify studies on predicting MVI in HCC through 18F-FDG PET/CT, from database inception to January 13, 2026. A combination of Medical Subject Headings (MeSH) with free-text terms was adopted, and the search strategy was adjusted according to the specifics of each database. The reference lists of the eligible studies were also examined for additional relevant literature. Search terms included ‘hepatocellular carcinoma’, ‘microvascular invasion’, ‘positron emission tomography’, and ‘fluorodeoxyglucose’. Detailed search strategies are presented in **Appendix 2**.

### 2.2 Eligibility criteria

#### 2.2.1 Inclusion criteria

(i) Participant: individuals were suspected of having HCC based on contrast-enhanced MRI, CT, or PET/CT before surgery, with postoperative pathology confirming the diagnosis; (ii) Exposure factor: a exposure group was defined as MVI-positive (MVI+), while a control group was defined as MVI-negative (MVI–); (iii) Outcome indicator: outcome indicators included odds ratios (ORs) for MVI associated with preoperative 18F-FDG PET/CT and its derived indices, area under the receiver operating characteristic curve (AUC), sensitivity, and specificity; (iv) Study design: only observational studies were included.

#### 2.2.2 Exclusion criteria

Studies meeting any of the conditions as follows were excluded: (i) individuals with a history of other malignant tumors; (ii) review articles, conference abstracts, letters, case reports, or similar publications; (iii) non-English studies; or (iv) studies with incomplete data.

### 2.3 Literature screening and data extraction

All retrieved records were uploaded to EndNote 21 to remove duplicates. Thereafter, two investigators independently screened the remaining studies against the eligibility criteria in a cross-checking manner. Any discrepancies between the two investigators were reconciled via discussion with a third researcher. A basic information extraction form was developed, and relevant data were extracted. The following information was collected from each study: (i) characteristics of included studies, including publication year, first author, and study location; (ii) clinical characteristics of affected patients, including sample size, sex, and age distribution; (iii) predictive performance indicators for radiomics-based models; and (iv) liver disease etiology, degree of tumor differentiation, and HCC stage; technical parameters and diagnostic threshold information, including PET/CT scanner model, imaging protocol, acquisition mode, reconstruction algorithm, SUV normalization method, and optimal cutoff value for maximum standardized uptake value (SUVmax).

### 2.4 Risk of bias assessment

Two researchers independently assessed risk of bias using Quality Assessment of Diagnostic Accuracy Studies-2 (QUADAS-2), a validated framework for diagnostic accuracy studies. The assessment mainly comprised ‘risk-of-bias judgment’ and ‘clinical applicability assessment’. The former is an evaluation of patient selection, index test, reference standard, and flow and timing, with each domain classified as ‘yes’, ‘no’, or ‘unclear’. The latter is primarily an assessment of the applicability of patient selection, index test, reference standard, and index condition, with each domain classified as ‘high’, ‘low’, or ‘unclear’ (7).

### 2.5 Statistical analysis

This meta-analysis was performed using software including Stata 15, MetaDisc 1.4, and MedCalc. OR was used as the effect size for the influencing factors, and its 95% confidence interval (CI) was provided. Heterogeneity was evaluated using the I² statistic and the Cochrane Q test. If I²≤50% or P>0.1, heterogeneity among studies was considered not significant, and a fixed-effects model was applied. Otherwise, a random-effects model was used to pool the predictive performance indicators, including pooled specificity, pooled sensitivity, pooled negative likelihood ratio (NLR), pooled positive likelihood ratio (PLR), pooled diagnostic odds ratio (DOR), and the summary receiver operating characteristic (SROC) curve with its AUC. An AUC of 0.5 indicates no predictive performance. An AUC between 0.5 and 0.7 represents poor predictive performance. An AUC between 0.7 and 0.9 signifies moderate predictive performance. An AUC between 0.9 and 1.0 denotes good predictive performance. When more than 10 studies were included, Deeks’ test was used to assess publication bias, with P<0.05 indicating a statistically significant difference.

### 2.6 Certainty of evidence assessment

The approach proposed by the Grading of Recommendations, Assessment, Development, and Evaluation (GRADE) Working Group for diagnostic accuracy studies was used to assess the certainty of evidence for the pooled sensitivity and specificity of each imaging indicator separately. Because diagnostic accuracy studies are designed to address questions of diagnostic accuracy, the initial certainty of evidence was rated as high; downgrading was subsequently judged across five domains: risk of bias, indirectness, inconsistency, imprecision, and publication bias. The certainty of evidence was ultimately classified into four levels: high, moderate, low, and very low. The GRADE evidence profile table was prepared using the diagnostic accuracy framework recommended by the GRADE Working Group (8) and generated with GRADEpro GDT.

## 3 Results

### 3.1 Literature screening

Initially, 326 records were identified, of which 134 duplicate records were excluded after screening. Following a thorough assessment based on titles and abstracts, 149 irrelevant records were removed. Of the 43 records retained for full-text assessment, 22 were eliminated for not meeting the inclusion criteria. Ultimately, 21 studies (1–4, 9–25) were included. A flow diagram for literature screening is presented in **Figure 1**.

**Figure 1:**
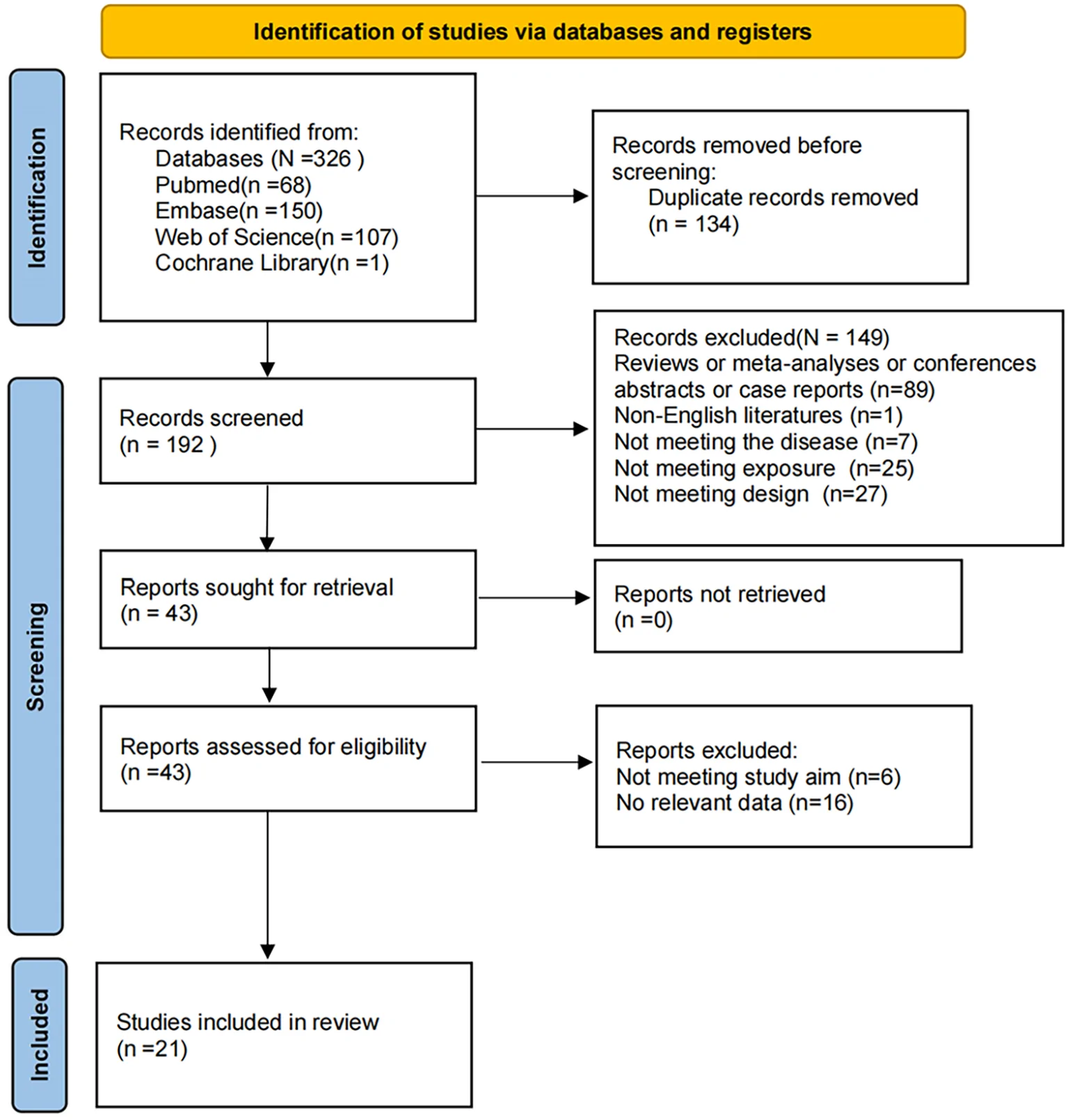
Flow diagram for literature screening.

### 3.2 Characteristics of included studies and risk of bias assessment

The study designs and patient characteristics of all included studies are illustrated in **Table 1** and **Table 2**. Among these, twenty were retrospective cohort studies, and one was a prospective cohort study. In total, 2,023 patients suffering from HCC were included, consisting of 890 MVI+ cases and 1133 MVI– cases. The indicators incorporated were as follows: anatomical and morphological features of tumors (tumor size); PET/CT-related metabolism parameters (SUVmax, mean standardized uptake value [SUVmean], peak standardized uptake value [SUVpeak], tumor-to-normal liver uptake ratio [TLR], metabolic tumor volume [MTV], total lesion glycolysis [TLG]); a radiomics parameter (Rad-score); and a subjective interpretation parameter (conventional visual assessment by PET/CT). QUADAS-2 was used to assess the quality of the 21 included studies. Overall, the applicability of the studies to the research question of this systematic review was favorable, with low risk of applicability concerns for ‘patient selection’, ‘index test’, and ‘reference standard’. The risk of bias was mainly concentrated in the domains of patient selection and reference standard. In the patient selection domain, 6 studies were judged to be at high risk and 12 at unclear risk. In the reference standard domain, 19 studies were rated to be at unclear risk. The overall quality of the index test and flow and timing domains was relatively good, with 15 and 13 studies, respectively, judged to be at low risk. Overall, the methodological quality of the included studies was acceptable. However, some studies had deficiencies in patient selection, blinding, and reporting of study procedures. Details are available in **Figure 2**.

**Table 1:** Characteristics of included studies.

Author	Year	Country	Study design	Group	Patient number	M/F	Age	FDG dose(Mbp)	Factors	Parameters
Wang TY et al	2024	China	R	MVI+/MVI-	62/51	MVI+:49/13 MVI-:43/8	MVI+:59.13 ± 9.86 MVI-:53.49 ± 11.4	370 MBq	1	2, 5
Jiang SP et al	2024	China	R	MVI+/MVI-	34/49	MVI+:23/11 MVI-:33/16	MVI+:>55(18) MVI-:>55(30)	3.7-5.5 MBq/kg	2, 5, 6	2, 5, 6, 7
Li Y et al	2025	China	R	MVI+/MVI-	87/44	MVI+:72/15 MVI-:36/8	NR	NR	NR	2, 3, 4, 5, 6, 7
Li YC et al	2021	China	R	MVI+/MVI-	36/44	MVI+:33/3 MVI-:40/4	MVI+:>50(22) MVI-:>50(30)	3.70-5.55MBq/kg	NR	NR
Shi HZ et al	2022	China	R	MVI+/MVI-	57/40	NR	NR	3.7 MBq/kg	NR	2, 3, 5
Wang YT et al	2022	China	R	MVI+/MVI-	38/59	NR	NR	3.70-5.55 mbq/kg	NR	NR
Hyun SH et al	2018	Korea	R	MVI+/MVI-	76/82	MVI+:58/18 MVI-:63/19	MVI+:57.82 ± 11.71 MVI-:56.29 ± 9.05	5.5 MBq/kg	NR	2, 5
Ahn SY et al	2015	Korea	R	MVI+/MVI-	13/38	MVI+:10/3 MVI-:32/6	MVI+:50.6 ± 9.1 MVI-:52.4 ± 5.9	5.2 MBq/kg	NR	2, 3, 5
Wang JQ et al.	2025	China	R	MVI+/MVI-	100/77	MVI+:94/6 MVI-:68/9	MVI+:52.18 ± 10.84 MVI-:54.6 ± 9.23	3.7 MBq/Kg	2, 3, 4, 5, 6	2, 3, 5, 6
Wu F et al	2024	China	R	MVI+/MVI-	72/52	MVI+:65/7 MVI-:44/8	NR	3.7 MBq/Kg	1, 2, 3, 4, 5, 6, 7	2, 3, 4, 5, 7
Çelebi F et al	2022	Turkey	R	MVI+/MVI-	16/28	MVI+:13/3 MVI-:23/5	MVI+:60 ± 6.78 MVI-:58.66 ± 13.67	4.54 ± 1MBq/kg	1, 2, 3, 4, 5, 6, 7	2
Cheung T To et al	2011	China	R	MVI+/MVI-	29/29	49/9	59.86 ± 11.52	370–550 MBq	NR	NR
Fukushima R et al	2024	Japan	R	MVI+/MVI-	40/89	108/21	71.1 ± 10.63	NR	7	2, 6, 7
Jiang CJ et al	2022	China	R	MVI+/MVI-	23/49	58/14	56 ± 9	3.7MBq/kg	1, 2, 3, 4, 5, 6, 7	2, 6, 7
Kobayashi T et al	2016	Japan	R	MVI+/MVI-	9/51	50/10	64.75 ± 10.81	3.7MBq/kg	2	2
Lim C et al	2019	France	P	MVI+/MVI-	26/52	63/15	66 ± 10	NR	NR	2, 5
Lv J et al	2021	China	R	MVI+/MVI-	24/10	29/5	53.8 ± 9.6	3.7MBq/kg	NR	2, 5
Yoh T et al	2021	Japan	R	MVI+/MVI-	64/155	NR	NR	NR	NR	5
Jiang CJ et al	2022	China	R	MVI+/MVI-	46/30	NR	NR	3.7MBq/kg	NR	2, 6, 7
Shirabe K et al	2014	Japan	R	MVI+/MVI-	23/40	MVI+:15/8 MVI-:19/21	MVI+:65 ± 14 MVI-:65 ± 9	NR	1, 2	2
Wu TH et al	2016	Japan	R	MVI+/MVI-	15/64	MVI+:9/6 MVI-:54/10	MVI+:73 ± 8.3 MVI-:68.9 ± 8.7	NR	NR	2

NR, not reported; R, retrospective study; P, prospective study. 1. Tumor size; 2. SUVmax (maximum standard uptake value); 3. SUVmean (mean standard uptake value); 4. SUVpeak (peak standard uptake value); 5. TLR/SUVmaxT/L (ratio of maximum standard uptake value in tumor to normal liver); 6. TLG (total lesion glycolysis;); 7. MTV (metabolic tumor volume).

**Table 2:** Characteristics of the included studies.

Author	Year	Country	Liver disease etiology	Tumor differentiation grade	HCC stage	PET/CT scanner types	Fasting/Blood glucose/dose/uptake time	Acquisition mode	Reconstruction algorithms	SUV normalisation	SUVmax cutoff value
Wang TY et al	2024	China	HBV	NR	NR	Siemens Biograph mCT 64	≥6h/<7 mmol/L/370 MBq/60min	3D; 2.5 min/bed; 5–6 bed	NR	NR	NR
Jiang SP et al	2024	China	Hepatitis	Well differentiated	BCLC stage (C-D)	Siemens Biograph mCT	≥6h/<11.0 mmol/L/3.7–5.5 MBq/kg/60min	3D	trueX and time-of-flight algorithm	Body weight normalisation	11.1
Li Y et al	2025	China	HBV/HCV	NR	NR	Siemens Biograph mCT 64	>6h/<7.1 mmol/L/NR/60min	3D; 2 min/bed; 6–7 bed	NR	Lean body mass normalisation	NR
Li YC et al	2021	China	HBV	Moderately/Poorly differentiated	BCLC stage (0-A)	GE Discovery ST 8	≥4h/3.6-6.1 mmol/L/3.70–5.55 MBq/kg/60min	NR	NR	Body weight normalisation	NR
Shi HZ et al	2022	China	HBV	NR	NR	SIEMENS Biograph mCT Flow	≥6h/NR/3.7 MBq/kg/60min	3D; 1.7 min/bed	2 iterations,21 subsets	NR	5.65
Wang YT et al	2022	China	NR	NR	NR	Siemens Biograph mCT 64	>6h/<11.1 mmol/L/3.70–5.55 MBq/kg/60min	3D; 2.5 min/bed; 5–6 bed	NR	NR	NR
Hyun SH et al	2018	Korea	NR	NR	BCLC stage (0-A)	GE Discovery STe/Siemens Biograph TruePoint/Siemens Biograph Duo/Philips Gemini TF16	≥6h/<140 mg/dL/5.5 MBq/kg, 6.0 MBq/kg, 333 MBq/60min	3D; 2–3 min/bed	2 iterations/20 subsets, 4 iterations/8 subsets, 2 iterations/8 subsets, 3 iterations/33 subsets	NR	NR
Ahn SY et al	2015	Korea	HBV/HCV/Alcohol	NR	NR	Siemens Biograph TruePoint/Siemens Biograph mCT40	≥6h/<200 mg/dL/5.2 MBq/kg/60min	3D; 1 min/bed; 7–9 bed	NR	NR	NR
Wang JQ et al.	2025	China	NR	Well/Moderately/Poorly differentiated	T-stage	GE Discovery PET/CT 710	NR/NR/3.7 MBq/kg/60min	3D; 2–3 min/bed; 6–7 bed	2 iterations,24 subsets	NR	NR
Wu F et al	2024	China	NR	Moderately/Poorly differentiated	T-stage	GE Discovery PET/CT 710	NR/NR/3.7 MBq/kg/60min	3D; 2–3 min/bed; 6–7 bed	2 iterations,24 subsets	NR	NR
Çelebi F et al	2022	Turkey	HBV/HCV/Alcohol	Well/Moderately/Poorly differentiated	NR	Siemens Biograph mCT	≥6h/<140 mg/dL/4.54 ± 1 MBq/kg/60min	NR	NR	Lean body mass normalisation	5.85
Cheung T To et al	2021	China	HBV/HCV	Poorly differentiated	TNM	GE Discovery ST 8	NR/NR/3.70-5.55 MBq/kg/60min	NR	NR	NR	NR
Fukushima R et al	2024	Japan	HBV/HCV	Poorly differentiated	BCLC stage (0-A)	GE Discovery STE/Siemens Biograph mCT 64	≥6h/<120 mg/dl/NR/NR	NR	NR	Body weight normalisation	NR
Jiang CJ et al	2022	China	HBV	Well/Moderately/Poorly differentiated/Undifferentiated	NR	Siemens Biograph 16 HR	≥6h/≤7.78 mmol/L/3.7 MBq/kg/60min	3D; 2 min/bed; 6–7 bed	NR	Body weight normalisation	4.5
Kobayashi T et al	2016	Japan	HBV/HCV/NBNC	Well/Moderately/Poorly differentiated	TNM	Siemens Biograph mCT	≥4h/<150 mg/dL/3.7 MBq/kg/60min	3D; 1.5–6 min/bed	NR	Body weight normalisation	3.2
Lim C et al	2019	France	HBV/HCV/Metabolic syndrome/Alcohol/Other/None	Poorly differentiated	NR	Philips Gemini GXL 16/Gemini TF 16	NR	NR	NR	NR	NR
Lv J et al	2021	China	NR	Well/Poorly differentiated	NR	GE Discovery VCT/uMI510/uMI780	≥6h/NR/3.7 MBq/kg/60min	3D; 2 min/bed	NR	NR	4.6
Yoh T et al	2021	Japan	HBV/HCV	Poorly differentiated/Undifferentiated	NR	GE Discovery STE	>4h/NR/NR/50min	3D; 3 min/bed; 5–6 bed	3 iterations,16 subsets	Body weight normalisation	NR
Jiang CJ et al	2022	China	NR	NR	T-stage	Siemens Biograph 16 HR	6h/<7.78 mmol/L/3.7 MBq/kg/60min	3D; 3 min/bed; 6–7 bed	NR	Body weight normalisation	NR
Shirabe K et al	2014	Japan	NR	Well/Moderately/Poorly differentiated	NR	GE Discovery ST Elite/Siemens Biograph mCT	≥4h/NR/NR/60min	3D; 3 min/bed	2 iterations/28 subsets,2 iterations/21 subsets	NR	4.2
Wu TH et al	2016	Japan	HBV/HCV	Well/Moderately/Poorly differentiated	NR	GE Discovery STE	>4h/NR/NR/50min	3D; 3 min/bed; 5–6 bed	3 iterations,16 subsets	Body weight normalisation	NR

**Figure 2:**
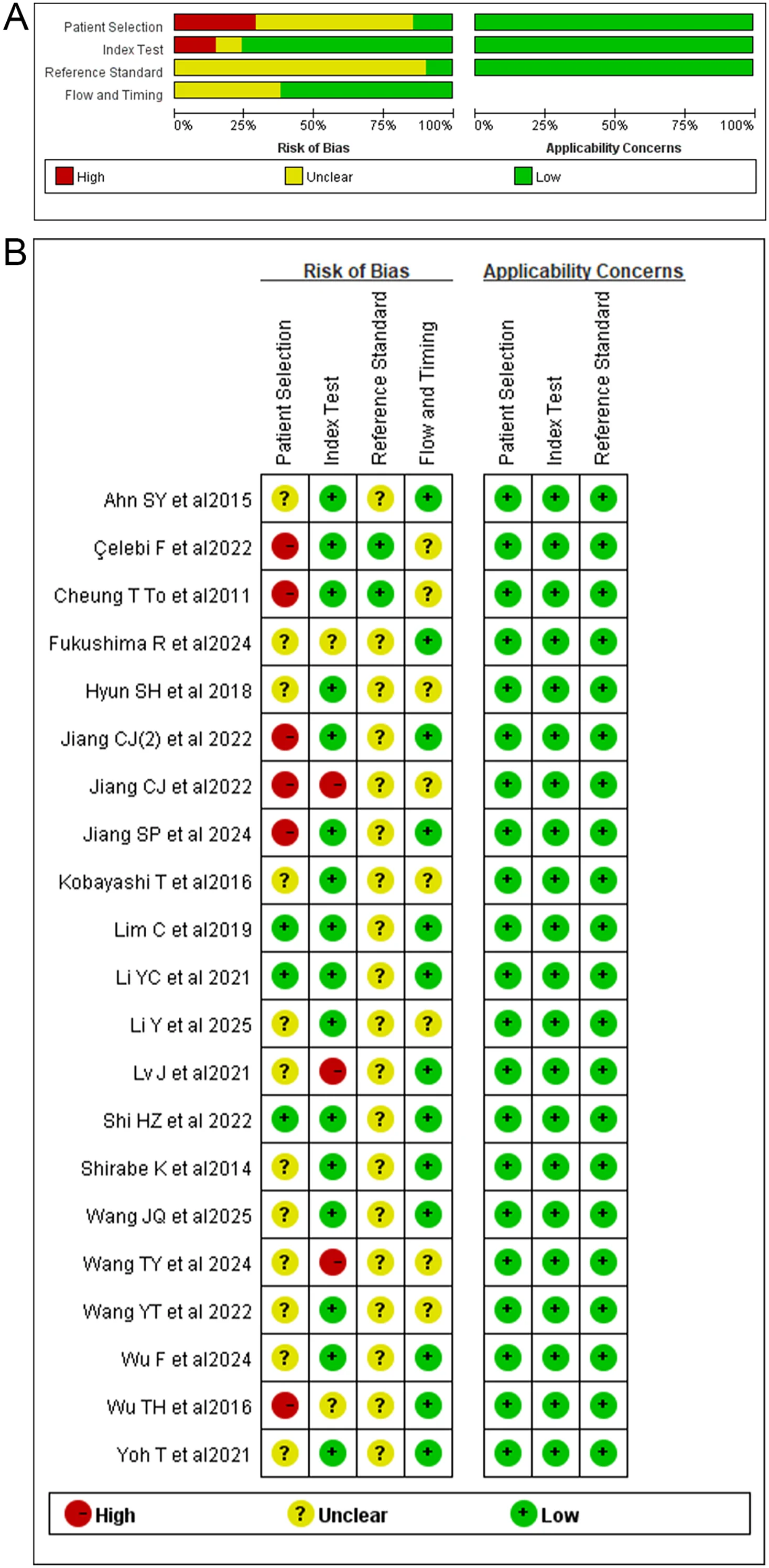
**(A)** A Methodological quality graph; **(B)** B Methodological quality summary.

### 3.3 Meta-analysis results

#### 3.3.1 Analysis of influencing factors

In the pooled univariable analyses, four potential influencing factors were analyzed. Tumor size (OR = 1.578, 95%CI: 1.006–2.474, P = 0.047), TLG (OR = 1.002, 95%CI: 1.001–1.003, P < 0.001), TLR (OR = 2.577, 95%CI: 1.446–4.593, P = 0.001), and SUVmax (OR = 1.272, 95%CI: 1.036–1.561, P = 0.021) were all significantly associated with an increased risk of MVI in HCC. However, after adjustment for confounding factors, a clear divergence from the above association pattern was observed: only SUVmax and TLR retained independent predictive performance. Specifically, the pooled multivariable OR for SUVmax increased to 2.140 (95%CI: 1.244–3.681, P = 0.006), which suggested that tumor metabolic activity was a robust independent predictor for MVI after adjustment for other confounders. The multivariable analysis for TLR was derived from only a single study (OR = 3.895, 95%CI: 1.178–12.877, P = 0.026). Although the effect size appeared large, this finding needed to be interpreted cautiously due to a lack of replicated validation. Of note, in the multivariable analysis for TLG, the OR was 0.999 (95%CI: 0.998–1.000, P = 0.016). Although statistically significant, the effect size was extremely close to the null value. SUVmean, SUVpeak, and MTV showed no statistically significant association with MVI in either univariable or multivariable analyses (**Figures 3**, **4**; **Table 3**; **Appendix 3**).

**Figure 3:**
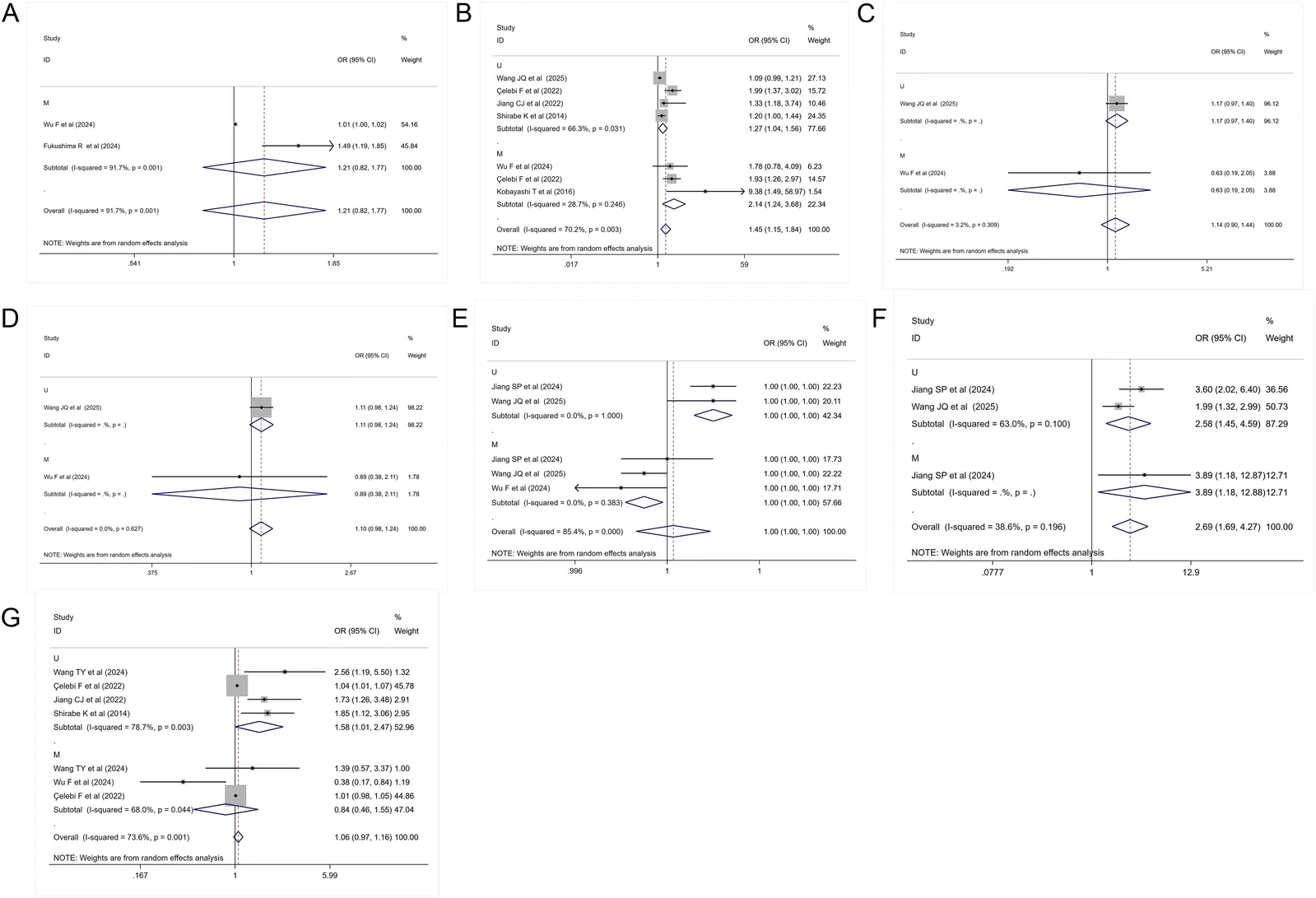
Subgroup analysis among different influencing factors for **(A)** MTV, **(B)** SUVmax, **(C)** SUVmean, **(D)** SUVpeak, **(E)** TLG, **(F)** TLR, **(G)** Tumor size. MTV, metabolic tumor volume; SUVmax, maximum standardized uptake value; SUVmean, mean standardized uptake value; SUVpeak, peak standardized uptake value; TLG, total lesion glycolysis; TLR, tumor-to-normal liver uptake ratio.

**Figure 4:**
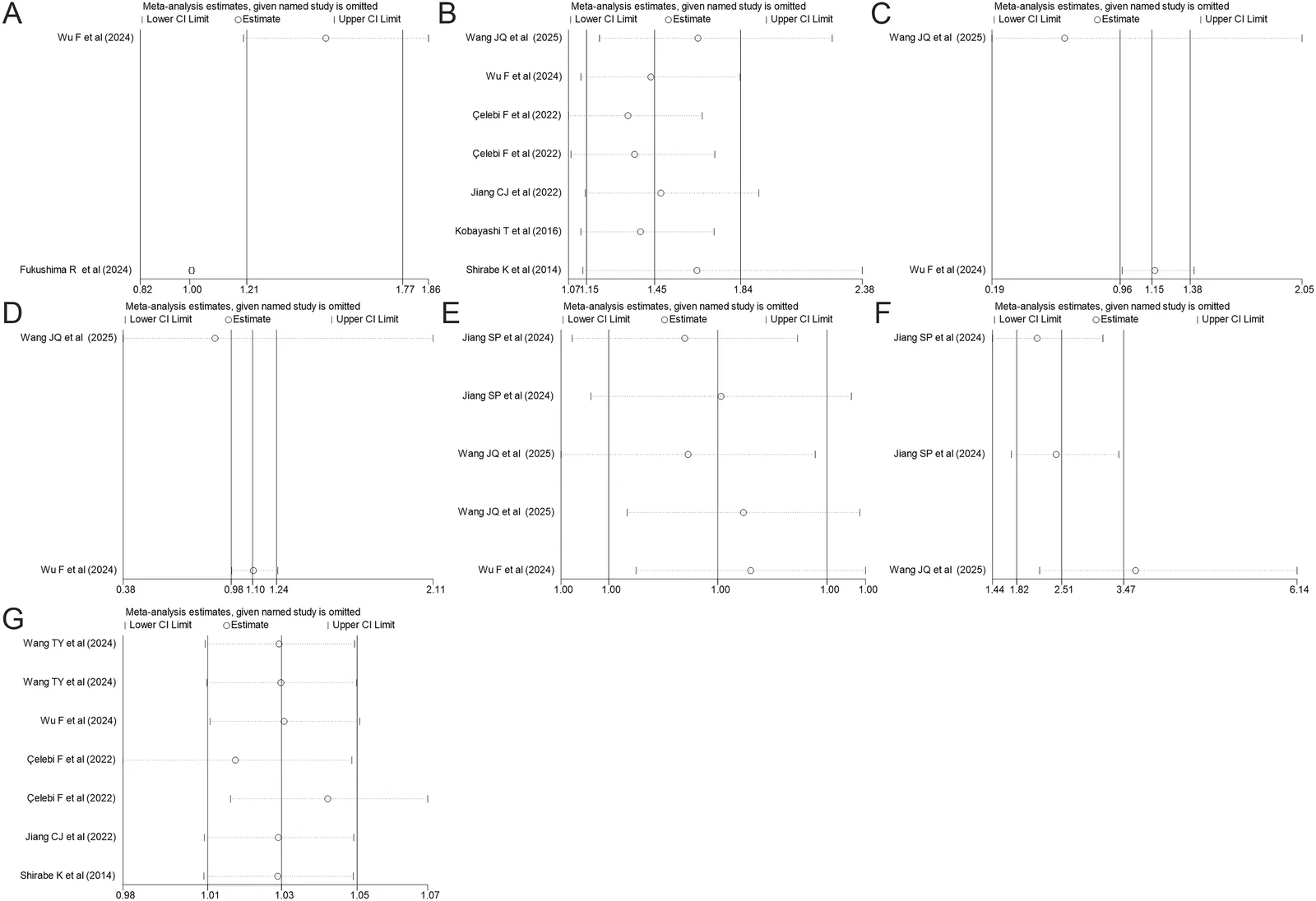
Sensitivity analysis among different influencing factors for **(A)** MTV, **(B)** SUVmax, **(C)** SUVmean, **(D)** SUVpeak, **(E)** TLG, **(F)** TLR, **(G)** Tumor size.

**Table 3:** Results from univariable and multivariable analyses.

Factor	Regression model	Number of studies	Heterogeneity test		Model	Meta-analysis results		
			P	I^2^/%		OR	95% CI	P
Tumor size	U	4	0.003	78.7	REM	1.578	1.006∼2.474	0.047
	M	3	0.044	68	REM	0.839	0.455∼1.548	0.575
MTV	M	2	0.001	91.7	REM	1.206	0.824∼1.765	0.334
TLG	U	2	1.000	0	FEM	1.002	1.001∼1.003	<0.001
	M	3	0.383	0	FEM	0.999	0.998∼1.000	0.016
TLR (SUVmaxT/L)	U	2	0.100	63	REM	2.577	1.446∼4.593	0.001
	M	1	/	/	/	3.895	1.178∼12.877	0.026
SUVmax	U	4	0.031	66.3	REM	1.272	1.036∼1.561	0.021
	M	3	0.246	28.7	FEM	2.140	1.244∼3.681	0.006
SUVmean	U	1	/	/	/	1.167	0.971∼1.402	0.099
	M	1	/	/	/	0.627	0.192∼2.048	0.440
SUVpeak	U	1	/	/	/	1.105	0.984∼1.241	0.093
	M	1	/	/	/	0.890	0.375∼2.112	0.792

#### 3.3.2 AUC analysis of imaging biomarkers for predicting MVI

The optimal discriminative ability was observed for SUVmax (AUC = 0.819, 95%CI: 0.739–0.899), with a relatively narrow CI, indicating the highest consistency and stability across studies. The radiomics-based Rad-score (AUC = 0.809, 95%CI: 0.762–0.857) was potentially effective for MVI prediction using texture features independent of subjective interpretation. SUVmean (AUC = 0.770, 95%CI: 0.649–0.890) and TLR (AUC = 0.742, 95%CI: 0.643–0.841) showed moderate predictive performance. Although the mean AUC values for SUVmean and TLR all exceeded 0.7, the lower bounds of their CIs approached 0.6, suggesting that their predictive performance might be at risk of attenuation when applied to external populations. In contrast, the AUC values for tumor size, MTV, and TLG were all below 0.7, indicating that the independent predictive efficacy of single morphology or volume parameters for MVI was limited (**Figure 5**; **Table 4**; **Appendix 4**).

**Figure 5:**
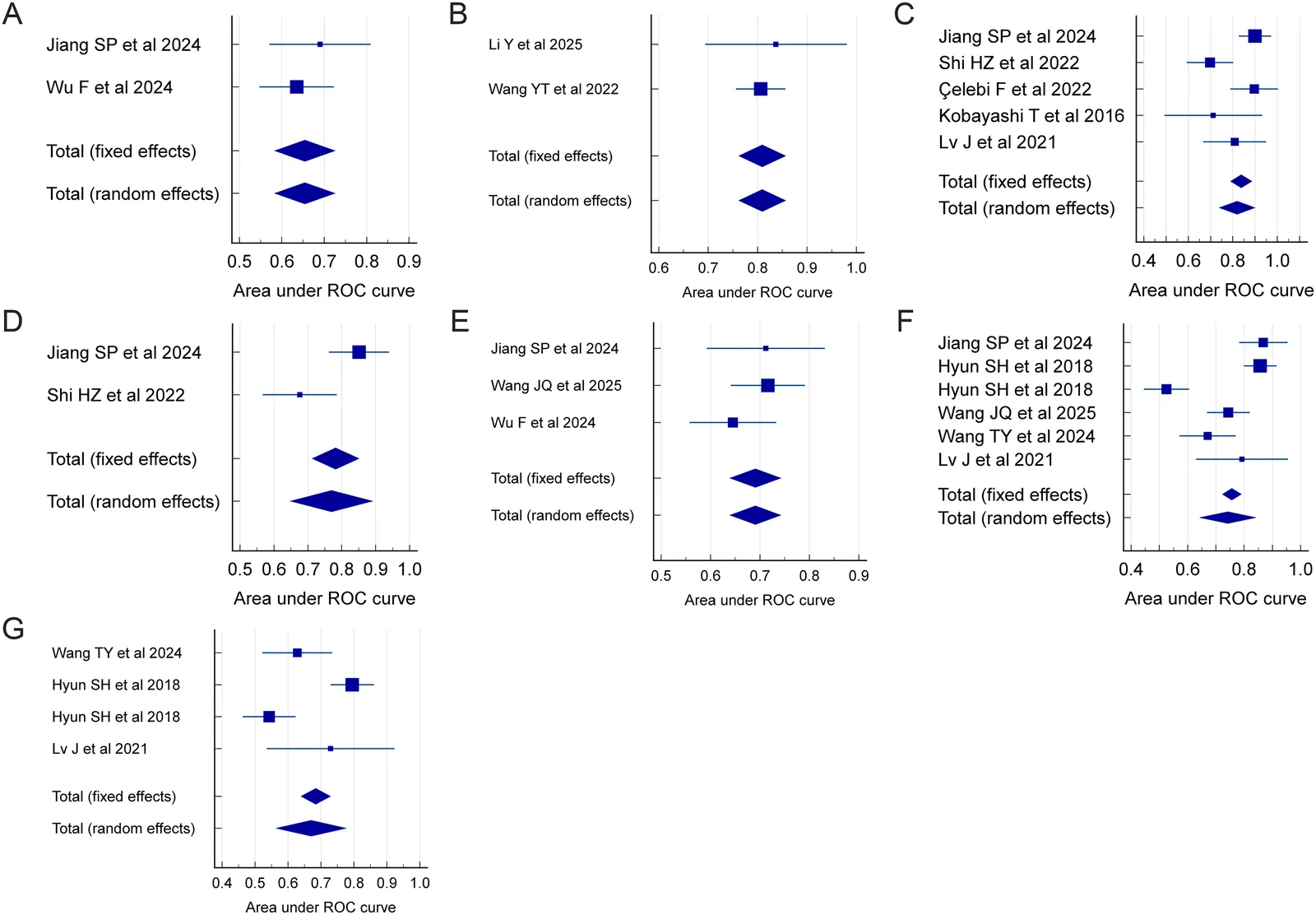
AUC values among different influencing factors for **(A)** MTV, **(B)** Rad-score, **(C)** SUVmax, **(D)** SUVmean, **(E)**TLG, **(F)**TLR, **(G)**Tumor size.

**Table 4:** Sensitivity, specificity, +LR, −LR, DOR, and AUC.

Factor	Sensitivity (95% CI)	Specificity (95% CI)	Diagnostic OR (95% CI)	+LR (95% CI)	−LR (95% CI)	AUC
Tumor size	0.56 (0.46, 0.65)	0.83 (0.74, 0.90)	7.50 (2.56, 21.98)	3.41 (2.41, 5.42)	0.47 (0.27, 0.83)	0.670 (0.564, 0.776)
MTV	0.67 (0.59, 0.74)	0.76 (0.69, 0.82)	8.21 (1.98, 33.97)	2.81 (1.65, 4.78)	0.36 (0.15, 0.87)	0.654 (0.583,0.725)
TLG	0.60 (0.42, 0.76)	0.84 (0.76, 0.90)	8.21 (4.75, 14.19)	3.86 (2.80, 5.32)	0.47 (0.31, 0.70)	0.691 (0.639, 0.742)
SUVmax	0.72 (0.64, 0.79)	0.80 (0.70, 0.87)	10.62 (5.12, 22.02)	3.66 (2.30, 5.84)	0.34 (0.25, 0.48)	0.819 (0.739, 0.899)
SUVmean	0.71 (0.61, 0.80)	0.79 (0.69, 0.87)	9.65 (4.52, 20.60)	3.42 (2.25, 5.19)	0.36 (0.22, 0.59)	0.770 (0.649, 0.890)
TLR	0.69 (0.54, 0.81)	0.81 (0.72, 0.87)	9.24 (4.95, 17.26)	3.56 (2.46, 5.14)	0.38 (0.26, 0.58)	0.742 (0.643, 0.841)
Rad-score	0.77 (0.68, 0.84)	0.76 (0.66, 0.84)	9.94 (4.47, 22.10)	3.06 (2.06, 4.54)	0.31 (0.21, 0.48)	0.809 (0.762, 0.857)
PET/CT diagnostic performance	0.75 (0.63, 0.84)	0.64 (0.51, 0.76)	5.17 (1.45, 18.41)	2.03 (1.14, 2.94)	0.39 (0.13, 1.20)	/

#### 3.3.3 Sensitivity, specificity, DOR, PLR, and NLR analyses of imaging biomarkers for predicting MVI

The pooled results demonstrated that both SUVmax and Rad-score exhibited relatively high sensitivity (0.72 and 0.77, respectively) and specificity (0.80 and 0.76, respectively). Except for conventional visual assessment by PET/CT, all other imaging biomarkers showed a pooled DOR exceeding 7.00, indicating that imaging assessments based on quantitative parameters overall were effective in discrimination. In terms of rule-in performance, the pooled PLR values for each biomarker fell within the range of 2.00 to 5.00, and the PLR values for TLG and SUVmax were relatively higher (3.86 and 3.66, respectively). However, the PLR values still indicated a moderate level of performance, suggesting that a positive result from a single imaging biomarker was insufficient to independently confirm MVI. Regarding rule-out performance, the pooled NLR values for SUVmax and Rad-score were 0.34 and 0.31, respectively, with the upper bounds of their CIs below 0.50. Together with the AUC analysis, SUVmax and the Rad-score were the biomarkers with the highest level of evidence and the most stable results currently. For conventional visual assessment by PET/CT, the CI for NLR crossed 1.00 (0.13–1.20), indicating suboptimal stability. This result suggested that in some study settings, a negative result from this method may not be able to rule out MVI, and clinical application should be cautious. In summary, based on the comprehensive analysis of diagnostic accuracy parameters, SUVmax and Rad-score were the most balanced and robust in terms of sensitivity, specificity, and NLR. This finding indicated that SUVmax and Rad-score hold high potential for non-invasive assessment of MVI preoperatively (**Table 4**; **Appendix 5**).

### 3.4 Certainty of evidence assessment

According to the GRADE assessment, the overall certainty of evidence for the diagnostic accuracy of the imaging parameters was low or very low. The certainty of evidence was low for the sensitivity of SUVmax and very low for its specificity. For SUVmean and radiomics score (Rad-score), the certainty of evidence was low for both sensitivity and specificity. The certainty of evidence was very low for the sensitivity of MTV, TLR, and tumor size and low for their specificity. The certainty of evidence was very low for both the sensitivity and specificity of TLG and PET-based overall assessment. The primary reasons for downgrading the certainty of evidence for each parameter were risk of bias, imprecision, and, for some parameters, substantial between-study inconsistency. For Rad-score, both sensitivity and specificity were downgraded to low-certainty evidence because of risk of bias and imprecision. Details are presented in **Appendix 6**.

## 4 Discussion

In this study, the efficacy of imaging biomarkers related to 18F-FDG PET/CT for preoperatively predicting MVI in HCC was systematically assessed through a meta-analysis. The results showed that SUVmax and Rad-score exhibited the most robust predictive performance, and they may be complementary in the dimensions of metabolic activity and texture heterogeneity. In contrast, the multivariable effect size of TLG approached the null line, and the cross-study variability of conventional visual assessment by PET/CT was relatively substantial.

This study indicated that SUVmax demonstrated moderate predictive performance in both univariable and multivariable analyses and was the most balanced and robust parameter in terms of sensitivity, specificity, and NLR. Çelebi et al. (16) have reported in 44 patients with HCC undergoing 18F-FDG PET/MRI that a multivariable analysis identifies SUVmax as the only independent predictor for MVI. With a cutoff value of 5.85, SUVmax showed high predictive efficacy as indicated by the AUC, sensitivity, and specificity. The study by Çelebi et al. has further validated that SUVmax retains independent predictive power after adjustment for other confounders such as tumor size and ADC value, which is consistent with the multivariable analysis conclusion of this study. Wang et al. (4) have also confirmed the independent predictive value of tumor metabolic activity in a PET/CT cohort of 113 patients, and a multivariable analysis has shown that active tumor metabolism (defined as TLR > 2) was an independent risk factor for MVI. Of note, in the study by Wang et al., SUVmax did not reach statistical significance, whereas TLR did. This apparent discrepancy actually reflects the inherent limitation of SUVmax measurement being confounded by individual variability in background liver metabolism (e.g., hepatitis activity, degree of cirrhosis, and blood glucose level). By introducing TLR for normalization, Wang et al. have successfully extracted an independent signal of relative tumor metabolic activity. This suggests that TLR and SUVmax are essentially two expressions of the same metabolism dimension. However, it should be noted that this conclusion was derived from single-center, retrospective data, and its generalizability needs to be validated based on multicenter data.

Lee et al. (6) have published a study on FDG PET/CT radiomics, reporting that both radiomics and semi-quantitative analysis (e.g., SUVmax) exhibited high predictive performance for MVI, with no statistically significant difference observed between the two. The study by Lee et al. and this study have produced almost identical point estimates, forming cross-validation, suggesting that at the current level of evidence, the discriminative ability of conventional semi-quantitative parameters and radiomics-based models for MVI prediction is substantially equivalent. Shi et al. (10) have performed a retrospective study based on single-center data in 97 patients with HCC, integrating PET radiomics, quantitative metabolism parameters, and multiparametric MRI to predict MVI, with patients randomly divided into a training set and a test set at a 7:3 ratio. In the training set, the AUC of the radiomics-based model exceeded 0.9; however, in the test set, the AUC dropped to 0.7. This notable drop between the training set and the validation set indicates that overfitting is observed in data processing, a phenomenon common in radiomics, associated with a small sample size, high feature dimensionality, and lack of external validation. Shi et al. have further constructed a hybrid model integrating radiomics classification results, SUVmax, ADC value, MRI arterial phase enhancement pattern, and tumor margin morphology. The AUC value in the test set further increases and is markedly higher than that of the radiomics-based model alone. The possible reason for this is that SUVmax captures information from the metabolic activity dimension, while radiomics supplements information on internal tumor microstructure from the texture heterogeneity dimension. Accordingly, the two are complementary in terms of information dimension.

However, not all studies have supported the above conclusions. Kim et al. (5) have performed a meta-analysis summarizing 14 studies and have concluded that FDG PET/CT shows low sensitivity for predicting MVI and is not recommended for routine use to rule out MVI. The reason for this discrepancy may be as follows: the ‘F-18 FDG PET’ studies included in the meta-analysis by Kim et al. comprise both quantitative SUVmax analysis and subjective assessment such as visual diagnosis, whereas this study strictly distinguished SUVmax from visual diagnosis. Moreover, the literature search performed by Kim et al. ended in 2020, whereas this study incorporated multiple studies of high quality published after 2021, resulting in a larger and more up-to-date evidence base. Secondly, this study clearly distinguished between univariable and multivariable evidence levels for SUVmax and directly compared it with other metabolism parameters, whereas the analysis by Kim et al. did not perform a dedicated pooling of multivariable effect sizes. In short, the conclusion drawn by Kim et al. pertains to the efficacy of PET/CT as an entire examination modality, whereas the analysis in this study indicates that when SUVmax is extracted as an independent quantitative parameter and adjusted for confounders, its predictive performance is clear and robust. Gou et al. (26) have conducted a meta-analysis including 26 studies covering 3,539 individuals with solitary HCC, comparing the diagnostic performance of radiomics methods versus non-radiomics methods for preoperatively predicting MVI. Compared with the non-radiomics-based model, a decreasing trend is observed in the AUC for the radiomics-based model. The study by Gou et al. concludes that ‘non-radiomics methods outperform radiomics methods’. It should be noted that the ‘non-radiomics-based model’ in the study by Gou et al. is not a single conventional parameter (e.g., SUVmax and tumor size), but rather a composite model incorporating clinical features, tumor margin smoothness, presence of peritumoral enhancement, and related quantitative imaging parameters, which contains more dimensions of information than a radiomics-based model alone. A higher AUC value is to some extent expected. Additionally, the RQS scores for the radiomics studies incorporated into the meta-analysis by Gou et al. are generally low, and the authors have explicitly stated that the high heterogeneity should be interpreted with caution. Accordingly, the conclusion drawn by Gou et al. reflects the uneven quality of methodologies in current radiomics studies, rather than a lack of predictive value of radiomics features themselves.

The above evidence consistently indicates that SUVmax and Rad-score emphasize different aspects of prediction: SUVmax reflects the metabolic activity dimension, whereas Rad-score reflects the texture heterogeneity dimension, mechanistically suggesting that the two may provide complementary information. SUVmax reflects the maximal FDG uptake capacity of tumor cells, and a higher value signifies greater glycolysis activity in that region. High glycolysis activity drives the proliferation, migration, and epithelial-mesenchymal transition of tumor cells through the Warburg effect, providing a metabolism basis for the occurrence of MVI. In addition, SUVmax represents the most hypoxic and metabolically active subclone within the tumor, and these cells are typically the most invasive and most likely to breach the capsule and invade blood vessels. Hence, SUVmax reflects the invasive potential of the tumor from the metabolism dimension. Rad-score captures the complexity of the spatial arrangement of pixel grayscale values within the tumor. This process converts visually imperceptible pixel arrangement patterns into objective, reproducible numerical values, unaffected by the subjective experience of an image interpreter. A high Rad-score corresponds to high texture heterogeneity. When the internal structure of a tumor becomes more fragmented and disordered, more uneven stress is exerted on its capsule, and vascular invasion is more likely to occur. In contrast, the multivariable effect size for TLG approached the null line, and its independent predictive performance was limited.

Regarding TLG, although its pooled multivariable effect size was statistically significant, its clinical significance was negligible. A fundamental distinction needs to be clarified in this study: statistical significance is not equivalent to clinical importance. P-value is largely a product of sample size, whereas effect size reflects clinical significance; when a sample size is sufficiently large, even a truly negligible effect may be deemed ‘significant’ by statistical testing, which explains why the per-unit effect size of TLG approaches one. However, it should be emphasized that this near-null per-unit result should not be interpreted as evidence that TLG itself lacks clinical value. From a biological perspective, TLG integrates MTV, which reflects tumor size and burden, with SUVmean, which reflects mean glycolytic intensity, and therefore characterizes total tumor metabolism more comprehensively than SUVmax. Larger and more metabolically active HCCs are more prone to MVI, and MVI is itself a hallmark of invasive tumor biology; thus, the dimension of ‘tumor load × invasive metabolism’ captured by TLG is mechanistically directly related to the occurrence of MVI. As a basic parameter on which TLG depends, SUVmean did not show independent predictive value in multivariable analysis. Because of the limited number of included studies and the lack of cirrhosis-stratified information, the performance of SUVmean in cirrhosis versus non- cirrhosis subgroups could not be examined. However, the fact that SUVmean is insufficient as a standalone predictor of MVI does not mean that it lacks clinical significance. SUVmax describes only the single most metabolically active point of a tumor, whereas SUVmean describes the average metabolic activity of the entire tumor. For evaluating total tumor metabolic load, staging, and treatment-response monitoring, SUVmean provides global information that SUVmax does not offer. Moreover, SUVmean captures intralesional metabolic heterogeneity, reflecting the integrated information of tumor cell metabolism and the microenvironment, such as necrosis and fibrosis. Necrosis is a feature of rapidly growing, invasive tumors, and a fibrotic background is a common substrate for HCC; the issue is therefore not that SUVmean is invalid, but that the absolute SUVmean signal is confounded when background correction is not applied. Cirrhotic liver tissue itself has relatively high background glucose metabolism, which may dilute or confound the tumor-specific SUVmean signal and thereby weaken its predictive ability. Among the individual parameters, the evidence was weakest for TLR. Its multivariable results were derived from only one primary study; accordingly, effect-size pooling was not feasible, and its independent predictive value at the summary level could not be assessed. This gap may reflect the still-limited application of TLR in the relevant literature, as well as the lack of standardization in TLR delineation and calculation. Accordingly, the generalizability of TLR as an independent risk factor should be interpreted with caution. Although the evidence for TLR was insufficient to support quantitative conclusions, the approach of using a ‘tumor/background ratio’ to predict invasiveness is consistent with the direction of recent findings involving FAPI tracers. FAPI-TBR similarly quantifies a tumor as a ‘tumor SUV/background SUV’ ratio, with higher TBR associated with greater malignancy and invasiveness. The difference between the two lies in their molecular substrates: FDG-TLR reflects glucose metabolism in the tumor cells themselves, whereas FAPI-TBR reflects activation of cancer-associated fibroblasts in the tumor stroma. This distinction leads to two practical consequences: FDG has an inherent blind spot for well-differentiated HCC (glucose-6-phosphatase activity leading to low uptake). In contrast, FAPI uptake is less dependent on differentiation and offers a lower background and higher contrast. FDG-TLR is susceptible to interference from high background metabolism in the setting of cirrhosis, whereas FAPI, owing to the extremely low background of normal liver, is relatively less affected by cirrhosis.

Beyond the performance of the individual biomarkers themselves, the pooled results from this study were also influenced by several methodological factors, among which the handling of tumor size was particularly prominent. The meta-analysis of tumor size showed substantial heterogeneity, which may be explained at three levels. First, the analysis approaches were inconsistent: some studies included tumor size as a continuous variable, whereas others included it as a dichotomous variable, and this difference in the scale of the effect measure was the primary source of heterogeneity. Second, the tumor-stage compositions differed markedly across studies: tumors in liver transplantation candidates were generally larger and at more advanced stages, and the association between tumor size and MVI was stronger in these patients, whereas the range of tumor-size variation in patients with small HCC was narrower, correspondingly attenuating the association. Third, the variables adjusted for in multivariable models differed across studies, rendering the net effect of tumor size incomparable. Technical differences were also important. Substantial heterogeneity was detected in the included studies in terms of scanner model, imaging protocol, acquisition mode, reconstruction algorithm, and SUV normalization method, with effects propagating along the chain of ‘technical parameters → absolute SUV scale → cutoff value → reproducibility of conclusions’. Three major vendors (Siemens, GE, and Philips) were represented, and several studies used multiple scanner models internally; consequently, SUVmax values across vendors were subject to systematic bias. Fasting duration, blood glucose thresholds, injection dosage, and imaging time also varied across studies. Among these factors, elevated blood glucose competes with FDG for glucose transport and may lower SUV, whereas prolonged imaging time increases FDG accumulation and consequently raises SUV. Although all studies used 3D acquisition, bed speed and the number of bed positions varied, affecting the stability of SUVmax measurements by altering count statistics and image noise. With respect to reconstruction, reconstruction incorporating a point-spread function and time of flight (true X+TOF) may substantially increase SUVmax compared with conventional OSEM, while the number of iterations and subsets used in OSEM also varied across studies. The mixed use of body weight normalization and lean body mass normalization further reduced the cross-study comparability of absolute SUV. These factors mainly affect the absolute SUV scale, thereby determining the cutoff value and influencing any pooled conclusion based on absolute SUV. The above statements are qualitative inferences based on technical principles and reporting characteristics. Because of incomplete reporting of key parameters and insufficient sample sizes within subgroups, these inferences could not yet be confirmed by quantitative subgroup analysis. At the level of study design, the vast majority of the included studies were single-center and retrospective; therefore, they carried a risk of selection bias. Selection bias is indeed a systematic feature of diagnostic research in this field: because MVI is only confirmed by postoperative pathology, any study of preoperative predictors necessarily retrospectively assembles cohorts of patients undergoing surgery and having complete pathological data. Prospective validation represents a subsequent stage of the evidence chain and remains insufficient in this field, which precisely reinforces the call for prospective multicenter validation made in the conclusion of this study. In addition, the included evidence was geographically concentrated, with the vast majority of studies originating from East Asia. This distribution was not attributable to the search or screening strategy; rather, it reflects the fact that most HCC cases worldwide occur in East Asia, with China representing the principal region for both new cases and deaths globally. This evidence structure indeed limits the generalizability of our findings. The current evidence derives predominantly from HBV-dominated Asian populations and underrepresents HCV-, alcohol-, and NAFLD-related HCC, whereas HCC of different etiologies may differ systematically in FDG uptake and invasiveness. For instance, NAFLD-related HCC frequently arises in a non-cirrhosis or mild fibrosis background, and some such tumors exhibit lower metabolic activity and superior differentiation, which may systematically alter the diagnostic threshold and sensitivity of SUVmax. Conversely, HBV-related HCC is associated with a higher incidence rate of MVI and greater invasiveness. The optimal SUVmax cutoff values varied across the included studies and were derived mainly from Chinese cohorts; therefore, directly extrapolating any single threshold to Western populations carries substantial risk.

Compared with previous relevant studies, this study is superior in the following aspects. First, by pooling multivariable-adjusted ORs, this study answered the question that is closer to clinical decision-making: whether PET parameters still provide incremental predictive value after adjustment for other known risk factors, and the result showed that SUVmax remained an independent risk factor following confounder adjustment. Second, this study clearly distinguished parameters such as SUVmax, TLR, MTV, and TLG, and compared their robustness differences. This refined analysis revealed the reason for the unstable effect size for parameters such as TLG due to heterogeneity in delineation thresholds, thereby providing more guidance for parameter selection in clinical settings. Additionally, this study included several new articles published after 2025. Li et al. (3) have constructed a combined nomogram integrating clinical features, PET metabolism parameters, and PET/CT radiomics features in 131 patients with HCC. In the validation set, the AUC for the combination model was markedly higher than that for the clinical model. This study confirmed, with the latest data, the conclusion drawn by Lee et al. that radiomics and semi-quantitative parameters have comparable performance. More significantly, the performance gain of the combination model in the study by Lee et al. directly supports the argument proposed in this study regarding the complementarity of the dimensions of metabolic activity and texture heterogeneity. As a consequence, this study may provide new evidence-based support for constructing combined prediction strategies clinically.

Several limitations of this study should be acknowledged. First, most included studies were single-center and retrospective; therefore, they carried a risk of selection bias. The evidence for Rad-score was derived solely from diagnostic-accuracy analyses, as most included radiomics studies did not report the OR of Rad-score for MVI. Consequently, whether Rad-score provides incremental predictive value independent of other established risk factors after adjustment could not be assessed, and its level of evidence may therefore be weaker than that of SUVmax. The results from the multivariable analyses of TLR were based on a single primary study and lacked repeated validation in large, multicenter cohorts; therefore, the conclusion that TLR serves as an independent risk factor should be generalized cautiously. The RQS scores for the radiomics studies were generally low, and none of the included studies underwent external validation. Because of insufficient sample sizes, missing data, and inconsistent classification in most included studies, subgroup effect sizes were not reported according to stratification variables; consequently, subgroup analyses by liver disease etiology, tumor differentiation grade, HCC stage, or geographic region could not be conducted. With regard to technical parameters and diagnostic-threshold information, reporting of cutoff values for scanner models, imaging protocols, reconstruction algorithms, SUV normalization methods, and optimal SUVmax was generally incomplete and inconsistent across the included studies, with marked inter-study differences. In addition, this study did not pool the diagnostic performance of combined models incorporating SUVmax and Rad-score; therefore, findings regarding their complementary value require support from external studies. Although the certainty of evidence was assessed using the approach proposed by the GRADE Working Group based on the available data, conclusions with low certainty should be interpreted with caution and further validated in prospective studies based on truly independent validation cohorts.

## 5 Conclusion

The available evidence supports SUVmax as one of the metabolic markers for MVI risk assessment in HCC preoperatively, with Rad-score similar to SUVmax in diagnostic discrimination. The complementary value of the two markers was not demonstrated by this Meta-analysis, but rather derived from inferences proposed by external studies. Moreover, because the included radiomics studies did not report a pooled effect size for the combined model integrating SUVmax and Rad-score, a Meta-analysis targeting this model is currently not feasible. Therefore, the complementarity of SUVmax and Rad-score should be regarded as a hypothesis to be tested. In the future, multicenter, prospective radiomics studies with rigorous external validation are warranted to further explore a combined prediction model based on SUVmax and Rad-score. On this basis, an integrated assessment workflow should be gradually established, by which preoperative decisions are guided.

## Data Availability

The original contributions presented in the study are included in the article/**Supplementary Material**. Further inquiries can be directed to the corresponding author.

## Appendix 1

PRISMA checklist.

## Appendix 2

Search strategy.

## Appendix 3

Analysis of influencing factors.

## Appendix 4

AUC analysis of imaging biomarkers for predicting MVI.

## Appendix 5

Sensitivity, specificity, DOR, PLR, and NLR analyses of imaging biomarkers for predicting MVI.

## Appendix 6

Certainty of Evidence Assessment.

## References

[1] Wu TH Hatano E Yamanaka K Seo S Taura K Yasuchika K . A non-smooth tumor margin on preoperative imaging predicts microvascular invasion of hepatocellular carcinoma. Surg Today. (2016) 46:1275–81. doi: 10.1007/s00595-016-1320-x 26983710

[2] Wu F Cao G Lu J Ye S Tang X . Correlation between 18 F-FDG PET/CT metabolic parameters and microvascular invasion before liver transplantation in patients with hepatocellular carcinoma. Nucl Med Commun. (2024) 45:1033–8. doi: 10.1097/mnm.0000000000001897 39267532 PMC11537472

[3] Li Y Liu J Lu D Wei L Ding E . The predictive value of (18)F-FDG PET/CT metabolic parameters and radiomics characteristics for microvascular invasion of hepatocellular carcinoma. Radiography (Lond). (2025) 31:103263. doi: 10.1016/j.radi.2025.103263 41372085

[4] Wang T Chen X Huang H Jia N . Early prediction of microvascular invasion (MVI) occurrence in hepatocellular carcinoma (HCC) by (18)F-FDG PET/CT and laboratory data. Eur J Med Res. (2024) 29:395. doi: 10.1186/s40001-024-01973-7 39080787 PMC11290007

[5] Kim K Kim SJ . Diagnostic test accuracies of F-18 FDG PET/CT for prediction of microvascular invasion of hepatocellular carcinoma: A meta-analysis. Clin Imaging. (2021) 79:251–8. doi: 10.1016/j.clinimag.2021.06.015 34157501

[6] Lee SW Jeong SY Kim SJ . Diagnostic performance of FDG PET/CT radiomics in predicting microvascular invasion in hepatocellular carcinoma compared to conventional metabolic parameters: a systematic review and meta-analysis. Ann Nucl Med. (2025) 39:1146–56. doi: 10.1007/s12149-025-02075-y 40581726

[7] Whiting PF Rutjes AW Westwood ME Mallett S Deeks JJ Reitsma JB . QUADAS-2: a revised tool for the quality assessment of diagnostic accuracy studies. Ann Intern Med. (2011) 155:529–36. doi: 10.7326/0003-4819-155-8-201110180-00009 22007046

[8] Schünemann HJ Mustafa RA Brozek J Steingart KR Leeflang M Murad MH . GRADE guidelines: 21 part 1. Study design, risk of bias, and indirectness in rating the certainty across a body of evidence for test accuracy. J Clin Epidemiol. (2020) 122:129–41. doi: 10.1016/j.jclinepi.2019.12.020 32060007

[9] Li Y Zhang Y Fang Q Zhang X Hou P Wu H . Radiomics analysis of [(18)F]FDG PET/CT for microvascular invasion and prognosis prediction in very-early- and early-stage hepatocellular carcinoma. Eur J Nucl Med Mol Imaging. (2021) 48:2599–614. doi: 10.1007/s00259-020-05119-9 33416951

[10] Shi H Duan Y Shi J Zhang W Liu W Shen B . Role of preoperative prediction of microvascular invasion in hepatocellular carcinoma based on the texture of FDG PET image: A comparison of quantitative metabolic parameters and MRI. Front Physiol. (2022) 13:928969. doi: 10.3389/fphys.2022.928969 36035488 PMC9412047

[11] Wang Y Luo S Jin G Fu R Yu Z Zhang J . Preoperative clinical-radiomics nomogram for microvascular invasion prediction in hepatocellular carcinoma using 18F-FDG PET/CT. BMC Med Imaging. (2022) 22:70. doi: 10.1186/s12880-022-00796-4 35428272 PMC9013080

[12] Hyun SH Eo JS Song BI Lee JW Na SJ Hong IK . Preoperative prediction of microvascular invasion of hepatocellular carcinoma using (18)F-FDG PET/CT: a multicenter retrospective cohort study. Eur J Nucl Med Mol Imaging. (2018) 45:720–6. doi: 10.1007/s00259-017-3880-4 29167923

[13] Ahn SY Lee JM Joo I Lee ES Lee SJ Cheon GJ . Prediction of microvascular invasion of hepatocellular carcinoma using gadoxetic acid-enhanced MR and (18)F-FDG PET/CT. Abdom Imaging. (2015) 40:843–51. doi: 10.1007/s00261-014-0256-0 25253426

[14] Wang J Kong X Cao G Ye S . Correlation study of 18F-FDG PET/CT metabolic parameters, heterogeneity index, and microvascular invasion, and its nomogram potential in predicting microvascular invasion in liver cancer before liver transplantation. Nucl Med Commun. (2025) 46:939–48. doi: 10.1097/mnm.0000000000002014 40551435 PMC12422608

[15] Jiang S Gao X Tian Y Chen J Wang Y Jiang Y . The potential of (18)F-FDG PET/CT metabolic parameter-based nomogram in predicting the microvascular invasion of hepatocellular carcinoma before liver transplantation. Abdom Radiol (NY). (2024) 49:1444–55. doi: 10.1007/s00261-023-04166-8 38265452

[16] Çelebi F Görmez A Ilgun AS Tokat Y Balcı NC . The value of 18F-FDG PET/MRI in prediction of microvascular invasion in hepatocellular carcinoma. Eur J Radiol. (2022) 149:110196. doi: 10.1016/j.ejrad.2022.110196 35144118

[17] Cheung TT Chan SC Ho CL Chok KS Chan AC Sharr WW . Can positron emission tomography with the dual tracers [11 C]acetate and [18 F]fludeoxyglucose predict microvascular invasion in hepatocellular carcinoma? Liver Transpl. (2011) 17:1218–25. doi: 10.1002/lt.22362 21688383

[18] Fukushima R Harimoto N Okuyama T Seki T Hoshino K Hagiwara K . New predictors of microvascular invasion for small hepatocellular carcinoma ≤ 3 cm. Int J Clin Oncol. (2024) 29:1182–90. doi: 10.1007/s10147-024-02553-9 38769190

[19] Jiang C Zhao L Xin B Ma G Wang X Song S . (18)F-FDG PET/CT radiomic analysis for classifying and predicting microvascular invasion in hepatocellular carcinoma and intrahepatic cholangiocarcinoma. Quant Imaging Med Surg. (2022) 12:4135–50. doi: 10.21037/qims-21-1167 35919043 PMC9338369

[20] Kobayashi T Aikata H Honda F Nakano N Nakamura Y Hatooka M . Preoperative fluorine 18 fluorodeoxyglucose positron emission tomography/computed tomography for prediction of microvascular invasion in small hepatocellular carcinoma. J Comput Assist Tomogr. (2016) 40:524–30. doi: 10.1097/rct.0000000000000405 26966955

[21] Lim C Salloum C Chalaye J Lahat E Costentin CE Osseis M . 18F-FDG PET/CT predicts microvascular invasion and early recurrence after liver resection for hepatocellular carcinoma: A prospective observational study. HPB (Oxford). (2019) 21:739–47. doi: 10.1016/j.hpb.2018.10.007 30401520

[22] Lv J Yin H Mao W Shi H . Investigating the value of pre-treatment (18)F-FDG PET/CT in predicting the pathological characteristic of hepatocellular carcinoma and recurrence after liver transplantation. Abdom Radiol (NY). (2021) 46:2490–7. doi: 10.1007/s00261-020-02872-1 33386905

[23] Yoh T Seo S Ogiso S Morino K Fukumitsu K Ishii T . Quantitative assessment of microvascular invasion in hepatocellular carcinoma using preoperative serological and imaging markers. HPB (Oxford). (2021) 23:1039–45. doi: 10.1016/j.hpb.2020.10.024 33262049

[24] Jiang C Ma G Liu Q Song S . The value of preoperative 18F-FDG PET metabolic and volumetric parameters in predicting microvascular invasion and postoperative recurrence of hepatocellular carcinoma. Nucl Med Commun. (2022) 43:100–7. doi: 10.1097/mnm.0000000000001478 34456318

[25] Shirabe K Toshima T Kimura K Yamashita Y Ikeda T Ikegami T . New scoring system for prediction of microvascular invasion in patients with hepatocellular carcinoma. Liver Int. (2014) 34:937–41. doi: 10.1111/liv.12459 24393295

[26] Gou J Li J Li Y Lu M Wang C Zhuo Y . The diagnostic accuracy between radiomics model and non-radiomics model for preoperative of microvascular invasion of solitary hepatocellular carcinoma: A systematic review and meta-analysis. Acad Radiol. (2024) 31:4419–33. doi: 10.1016/j.acra.2024.04.003 38664142

