# Supplementary material for: Imaging biomarkers related to 18F-FDG PET/CT for predicting microvascular invasion in hepatocellular carcinoma: a systematic review and meta-analysis
Source: Front Oncol. 2026 Oct 12;16:1948193. doi: 10.3389/fonc.2026.1948193 (PMC13696251; doi:10.3389/fonc.2026.1948193)
Supplement: Supplementary file 2 [file SupplementaryFile2.docx]

**Appendix 2** Search strategies

- PubMed

| Search number | Query | Sort By | Filters | Search Details | Results | Time | Date |
| --- | --- | --- | --- | --- | --- | --- | --- |
| 6 | #2 and #3 and #4 | Most Recent | | ("adult liver cancer*"[Title/Abstract] OR "carcinoma of the liver"[Title/Abstract] OR "hepat*"[Title/Abstract] OR "liver carcinoma*"[Title/Abstract] OR "malignant hepatoma"[Title/Abstract] OR "carcinoma, hepatocellular"[MeSH Terms]) AND ("Microvascular Invasion"[Title/Abstract] OR "MVI"[Title/Abstract]) AND ("Positron-Emission Tomography"[MeSH Terms] OR "Fluorodeoxyglucose F18"[MeSH Terms] OR ("18FDG"[Title/Abstract] OR "deoxyfluoroglucose"[Title/Abstract] OR "Fludeoxyglucose F 18"[Title/Abstract] OR "fluorodeoxyglucose"[Title/Abstract] OR "FDG"[Title/Abstract] OR "positron tomograph*"[Title/Abstract] OR "PET"[Title/Abstract])) | 68 | 4:48:26 | 2026/1/13 |
| 5 | ((("Adult Liver Cancer*"[Title/Abstract] OR "carcinoma in the liver"[Title/Abstract] OR "carcinoma of the liver"[Title/Abstract] OR "hepat*"[Title/Abstract] OR "liver carcinoma*"[Title/Abstract] OR "malignant hepatoma"[Title/Abstract]) OR ("Carcinoma, Hepatocellular"[Mesh])) AND ("Microvascular Invasion "[Title/Abstract] OR "MVI"[Title/Abstract])) AND ((("Positron-Emission Tomography"[Mesh]) OR ("Fluorodeoxyglucose F18"[Mesh])) OR ("18FDG"[Title/Abstract] OR "deoxyfluoroglucose"[Title/Abstract] OR "Fludeoxyglucose F 18"[Title/Abstract] OR "fluorodeoxyglucose"[Title/Abstract] OR "FDG"[Title/Abstract] OR "positron tomograph*"[Title/Abstract] OR "PET"[Title/Abstract])) | Most Recent | | ("adult liver cancer*"[Title/Abstract] OR "carcinoma of the liver"[Title/Abstract] OR "hepat*"[Title/Abstract] OR "liver carcinoma*"[Title/Abstract] OR "malignant hepatoma"[Title/Abstract] OR "carcinoma, hepatocellular"[MeSH Terms]) AND ("Microvascular Invasion"[Title/Abstract] OR "MVI"[Title/Abstract]) AND ("Positron-Emission Tomography"[MeSH Terms] OR "Fluorodeoxyglucose F18"[MeSH Terms] OR ("18FDG"[Title/Abstract] OR "deoxyfluoroglucose"[Title/Abstract] OR "Fludeoxyglucose F 18"[Title/Abstract] OR "fluorodeoxyglucose"[Title/Abstract] OR "FDG"[Title/Abstract] OR "positron tomograph*"[Title/Abstract] OR "PET"[Title/Abstract])) | 68 | 4:47:57 | 2026/1/13 |
| 4 | (("Positron-Emission Tomography"[Mesh]) OR ("Fluorodeoxyglucose F18"[Mesh])) OR ("18FDG"[Title/Abstract] OR "deoxyfluoroglucose"[Title/Abstract] OR "Fludeoxyglucose F 18"[Title/Abstract] OR "fluorodeoxyglucose"[Title/Abstract] OR "FDG"[Title/Abstract] OR "positron tomograph*"[Title/Abstract] OR "PET"[Title/Abstract]) | Most Recent | | "Positron-Emission Tomography"[MeSH Terms] OR "Fluorodeoxyglucose F18"[MeSH Terms] OR "18FDG"[Title/Abstract] OR "deoxyfluoroglucose"[Title/Abstract] OR "Fludeoxyglucose F 18"[Title/Abstract] OR "fluorodeoxyglucose"[Title/Abstract] OR "FDG"[Title/Abstract] OR "positron tomograph*"[Title/Abstract] OR "PET"[Title/Abstract] | 182,677 | 4:47:38 | 2026/1/13 |
| 3 | "Microvascular Invasion "[Title/Abstract] OR "MVI"[Title/Abstract] | Most Recent | | "Microvascular Invasion"[Title/Abstract] OR "MVI"[Title/Abstract] | 3,291 | 4:46:45 | 2026/1/13 |
| 2 | ("Adult Liver Cancer*"[Title/Abstract] OR "carcinoma in the liver"[Title/Abstract] OR "carcinoma of the liver"[Title/Abstract] OR "hepat*"[Title/Abstract] OR "liver carcinoma*"[Title/Abstract] OR "malignant hepatoma"[Title/Abstract]) OR ("Carcinoma, Hepatocellular"[Mesh]) | Most Recent | | "adult liver cancer*"[Title/Abstract] OR "carcinoma of the liver"[Title/Abstract] OR "hepat*"[Title/Abstract] OR "liver carcinoma*"[Title/Abstract] OR "malignant hepatoma"[Title/Abstract] OR "carcinoma, hepatocellular"[MeSH Terms] | 923,230 | 4:46:07 | 2026/1/13 |
| 1 | "Carcinoma, Hepatocellular"[Mesh] | Most Recent | | "carcinoma, hepatocellular"[MeSH Terms] | 117,901 | 4:45:33 | 2026/1/13 |

- Web of science

|  | Query | Results | Running date |
| --- | --- | --- | --- |
| - WOS.IC: 1993 to 2026 - WOS.CCR: 1985 to 2026 - WOS.SCI: 2000 to 2026 - WOS.AHCI: 2000 to 2026 - WOS.ESCI: 2021 to 2026 - WOS.SSCI: 2000 to 2026 | (Adult Liver Cancer*) OR (carcinoma in the liver) OR (carcinoma of the liver) OR (hepat*) OR (liver carcinoma*) OR (malignant hepatoma) (主题) AND (Microvascular Invasion ) OR (MVI) (主题) AND (18FDG) OR (deoxyfluoroglucose) OR (Fludeoxyglucose F 18) OR (fluorodeoxyglucose) OR (FDG) OR (positron tomograph*) OR (PET) (主题) | 107 | Tue Jan 13 2026 17:57:30 GMT+0800 (中国标准时间) |

- Embase

| No. | Query | Results | Date |
| --- | --- | --- | --- |
| #9 | #3 AND #7 AND #8 | 150 | 13-Jan-26 |
| #8 | 'microvascular invasion':ti,ab,kw OR 'mvi':ti,ab,kw | 5234 | 13-Jan-26 |
| #7 | #4 OR #5 OR #6 | 409426 | 13-Jan-26 |
| #6 | '18fdg':ti,ab,kw OR 'deoxyfluoroglucose':ti,ab,kw OR 'fludeoxyglucose f 18':ti,ab,kw OR 'fluorodeoxyglucose':ti,ab,kw OR 'fdg':ti,ab,kw OR 'positron tomograph*':ti,ab,kw OR 'pet':ti,ab,kw | 295939 | 13-Jan-26 |
| #5 | 'fluorodeoxyglucose'/exp | 19551 | 13-Jan-26 |
| #4 | 'positron emission tomography'/exp | 297448 | 13-Jan-26 |
| #3 | #1 OR #2 | 1345911 | 13-Jan-26 |
| #2 | 'adult liver cancer*':ti,ab,kw OR 'carcinoma in the liver':ti,ab,kw OR 'carcinoma of the liver':ti,ab,kw OR 'hepat*':ti,ab,kw OR 'liver carcinoma*':ti,ab,kw OR 'malignant hepatoma':ti,ab,kw | 1308884 | 13-Jan-26 |
| #1 | 'liver cell carcinoma'/exp | 253617 | 13-Jan-26 |

- cochrane

Search Name:

Date Run:	13/01/2026 17:56:14

Comment:

| ID | Search | Hits |
| --- | --- | --- |
| #1 | MeSH descriptor: [Carcinoma, Hepatocellular] explode all trees | 2876 |
| #2 | ('Adult Liver Cancer*' OR 'carcinoma in the liver' OR 'carcinoma of the liver' OR 'hepat*' OR 'liver carcinoma*' OR 'malignant hepatoma'):ti,ab,kw | 61266 |
| #3 | #1 OR #2 | 61266 |
| #4 | ('Microvascular Invasion ' OR 'MVI'):ti,ab,kw | 266 |
| #5 | MeSH descriptor: [Positron-Emission Tomography] explode all trees | 1959 |
| #6 | MeSH descriptor: [Fluorodeoxyglucose F18] explode all trees | 960 |
| #7 | ('18FDG' OR 'deoxyfluoroglucose' OR 'Fludeoxyglucose F 18' OR 'fluorodeoxyglucose' OR 'FDG' OR 'positron tomograph*' OR 'PET'):ti,ab,kw | 11810 |
| #8 | #5 OR #6 OR #7 | 11810 |
| #9 | #3 AND #4 AND #8 | 1 |
