# Supplementary material for: Imaging biomarkers related to 18F-FDG PET/CT for predicting microvascular invasion in hepatocellular carcinoma: a systematic review and meta-analysis
Source: Front Oncol. 2026 Oct 12;16:1948193. doi: 10.3389/fonc.2026.1948193 (PMC13696251; doi:10.3389/fonc.2026.1948193)
Supplement: Supplementary file 4 [file SupplementaryFile4.docx]

**Appendix 4** AUC Analysis of Imaging Biomarkers for Predicting MVI

MTV

Rad-score

SUVmax

SUVmean

TLG

TLR

Tumor size
