# Supplementary material for: Imaging biomarkers related to 18F-FDG PET/CT for predicting microvascular invasion in hepatocellular carcinoma: a systematic review and meta-analysis
Source: Front Oncol. 2026 Oct 12;16:1948193. doi: 10.3389/fonc.2026.1948193 (PMC13696251; doi:10.3389/fonc.2026.1948193)
Supplement: Supplementary file 5 [file SupplementaryFile5.docx]

**Appendix 5** Sensitivity, Specificity, DOR, PLR, and NLR Analyses of Imaging Biomarkers for Predicting MVI

MTV

|  |  |  |
| --- | --- | --- |
| DOR(2) | DOR | NLR |
|  |  |  |
| PLR NLR | PLR | ROC curve |
|  |  |  |
| Sensitivity Specificity | Sensitivity | Specificity |
|  |  |  |
| Threshold |  |  |

PET CT diagnostic performance

|  |  |  |
| --- | --- | --- |
| DOR (2) | DOR | NLR |
|  |  |  |
| PLR NLR | PLR | Sensitivity Specificity |
|  |  |  |
| Sensitivity | Specificity |  |

Rad-score

|  |  |  |
| --- | --- | --- |
| DOR (2) | DOR | NLR |
|  |  |  |
| PLR NLR | PLR | Sensitivity Specificity |
|  |  |  |
| Sensitivity | Specificity |  |

SUVmax

|  |  |  |
| --- | --- | --- |
| DOR | Fagan | PLR NLR |
|  |  |  |
| ROC curve | Sensitivity Specificity | Threshold |

SUVmean

|  |  |  |
| --- | --- | --- |
| DOR (2) | DOR | NLR |
|  |  |  |
| PLR NLR | PLR | Sensitivity Specificity |
|  |  |  |
| Sensitivity | Specificity |  |

TLG

|  |  |  |
| --- | --- | --- |
| DOR | Fagan | PLR NLR |
|  |  |  |
| ROC curve | Sensitivity Specificity | Threshold |

TLR

|  |  |  |
| --- | --- | --- |
| DOR | Fagan | PLR NLR |
|  |  |  |
| ROC curve | Sensitivity Specificity | Threshold |

tumor size

|  |  |  |
| --- | --- | --- |
| DOR(2) | DOR | NLR |
|  |  |  |
| PLR NLR | PLR | ROC curve |
|  |  |  |
| Sensitivity Specificity | Sensitivity | Specificity |
|  |  |  |
| Threshold |  |  |
