# Supplementary material for: Imaging biomarkers related to 18F-FDG PET/CT for predicting microvascular invasion in hepatocellular carcinoma: a systematic review and meta-analysis
Source: Front Oncol. 2026 Oct 12;16:1948193. doi: 10.3389/fonc.2026.1948193 (PMC13696251; doi:10.3389/fonc.2026.1948193)
Supplement: Supplementary file 6 [file SupplementaryFile6.docx]

**Appendix 6** Certainty of Evidence Assessment

**(1) Question**: Should Preoperative 18F-FDG PET/CT-derived **SUVmax** be used to diagnose Histopathologically confirmed microvascular invasion in hepatocellular carcinoma in Patients with hepatocellular carcinoma undergoing liver resection or transplantation?

| Outcome | № of studies (№ of patients) | Study design | Factors that may decrease certainty of evidence | | | | | Effect per 1,000 patients tested | Test accuracy CoE |
| --- | --- | --- | --- | --- | --- | --- | --- | --- | --- |
|  |  |  | Risk of bias | Indirectness | Inconsistency | Imprecision | Publication bias | pre-test probability of 39.6% |  |
| **True positives** (patients with Histopathologically confirmed microvascular invasion in hepatocellular carcinoma) | 7 studies 202 patients | cohort & case-control type studies | serious^a^ | not serious^b^ | not serious^c^ | serious^d^ | none^g^ | 285 (253 to 313) | ⨁⨁◯◯ Low^a,b,c,d^ |
| **False negatives** (patients incorrectly classified as not having Histopathologically confirmed microvascular invasion in hepatocellular carcinoma) |  |  |  |  |  |  |  | 111 (83 to 143) |  |
| **True negatives** (patients without Histopathologically confirmed microvascular invasion in hepatocellular carcinoma) | 7 studies 308 patients | cohort & case-control type studies | serious^a^ | not serious^b^ | serious^e^ | serious^f^ | none^g^ | 483 (423 to 525) | ⨁◯◯◯ Very low^a,b,e,f^ |
| **False positives** (patients incorrectly classified as having Histopathologically confirmed microvascular invasion in hepatocellular carcinoma) |  |  |  |  |  |  |  | 121 (79 to 181) |  |

#### Explanations

a. Serious risk of bias because two of the seven studies were judged to be at high risk of bias in patient selection, one study was at high risk in the index-test domain, and most studies provided insufficient information regarding blinding of the histopathological reference-standard assessment. Several studies also had unclear risk concerning flow and timing.

b. The population, index test, target condition and histopathological reference standard were generally consistent with the review question. One PET/MRI study was included, but it accounted for a small proportion of the evidence.

c. Not serious. Statistical heterogeneity in sensitivity was low to moderate (I²=26.1%), and the direction of results was generally consistent.

d. Serious imprecision because sensitivity was based on only 202 patients with MVI, and the 95% CI corresponded to 83–143 false-negative results per 1,000 patients tested.

e. Serious inconsistency because specificity showed substantial heterogeneity (I²=69.3%) across studies, possibly related to different SUVmax thresholds, imaging protocols and patient characteristics.

f. Serious imprecision because specificity was based on only 308 patients without MVI, and the 95% CI corresponded to 79–181 false-positive results per 1,000 patients tested.

g. Publication bias could not be reliably assessed because fewer than 10 studies were included in each biomarker analysis; therefore, no additional rating down was applied.

**(2) Question**: Should Preoperative 18F-FDG PET/CT-derived **SUVmean** be used to diagnose Histopathologically confirmed microvascular invasion in hepatocellular carcinoma in Patients with hepatocellular carcinoma undergoing liver resection or transplantation?

| Outcome | № of studies (№ of patients) | Study design | Factors that may decrease certainty of evidence | | | | | Effect per 1,000 patients tested | Test accuracy CoE |
| --- | --- | --- | --- | --- | --- | --- | --- | --- | --- |
|  |  |  | Risk of bias | Indirectness | Inconsistency | Imprecision | Publication bias | pre-test probability of 50.6% |  |
| **True positives** (patients with Histopathologically confirmed microvascular invasion in hepatocellular carcinoma) | 2 studies 91 patients | cohort & case-control type studies | serious^a^ | not serious | not serious^b^ | serious^c^ | none^e^ | 359 (309 to 405) | ⨁⨁◯◯ Low^a,b,c^ |
| **False negatives** (patients incorrectly classified as not having Histopathologically confirmed microvascular invasion in hepatocellular carcinoma) |  |  |  |  |  |  |  | 147 (101 to 197) |  |
| **True negatives** (patients without Histopathologically confirmed microvascular invasion in hepatocellular carcinoma) | 2 studies 89 patients | cohort & case-control type studies | serious^a^ | not serious | not serious^b^ | serious^d^ | none^e^ | 390 (341 to 430) | ⨁⨁◯◯ Low^a,b,d^ |
| **False positives** (patients incorrectly classified as having Histopathologically confirmed microvascular invasion in hepatocellular carcinoma) |  |  |  |  |  |  |  | 104 (64 to 153) |  |

#### Explanations

a. Serious risk of bias because one of the two contributing studies was at high risk of bias in patient selection, and both studies provided insufficient information regarding whether the histopathological reference-standard assessment was interpreted without knowledge of the index-test results.

b. Statistical heterogeneity was not considered serious. Sensitivity showed moderate heterogeneity (I²=42.8%), whereas specificity showed no important heterogeneity.

c. Serious imprecision because sensitivity was based on only 91 patients with MVI, and the 95% CI corresponded to 101–197 false-negative results per 1,000 patients tested.

d. Serious imprecision because specificity was based on only 89 patients without MVI, and the 95% CI corresponded to 64–153 false-positive results per 1,000 patients tested.

e. Publication bias could not be reliably assessed because fewer than 10 studies were included in each biomarker analysis; therefore, no additional rating down was applied.

**(3) Question**: Should Preoperative 18F-FDG PET/CT-derived **metabolic tumor volume (MTV)** be used to diagnose Histopathologically confirmed microvascular invasion in hepatocellular carcinoma in Patients with hepatocellular carcinoma undergoing liver resection or transplantation?

| Outcome | № of studies (№ of patients) | Study design | Factors that may decrease certainty of evidence | | | | | Effect per 1,000 patients tested | Test accuracy CoE |
| --- | --- | --- | --- | --- | --- | --- | --- | --- | --- |
|  |  |  | Risk of bias | Indirectness | Inconsistency | Imprecision | Publication bias | pre-test probability of 43.2% |  |
| **True positives** (patients with Histopathologically confirmed microvascular invasion in hepatocellular carcinoma) | 3 studies 145 patients | cohort & case-control type studies | serious^a^ | not serious | serious^b^ | serious^c^ | none^f^ | 289 (255 to 320) | ⨁◯◯◯ Very low^a,b,c^ |
| **False negatives** (patients incorrectly classified as not having Histopathologically confirmed microvascular invasion in hepatocellular carcinoma) |  |  |  |  |  |  |  | 143 (112 to 177) |  |
| **True negatives** (patients without Histopathologically confirmed microvascular invasion in hepatocellular carcinoma) | 3 studies 191 patients | cohort & case-control type studies | serious^a^ | not serious | not serious^d^ | serious^e^ | none^f^ | 432 (392 to 466) | ⨁⨁◯◯ Low^a,d,e^ |
| **False positives** (patients incorrectly classified as having Histopathologically confirmed microvascular invasion in hepatocellular carcinoma) |  |  |  |  |  |  |  | 136 (102 to 176) |  |

#### Explanations

a. Serious risk of bias because one of the three studies was at high risk of bias in patient selection, while the remaining studies had unclear concerns regarding patient selection, prespecification of the index-test threshold, reference-standard blinding, or flow and timing.

b. Serious inconsistency because sensitivity varied substantially across studies (I²=89.7%), probably due to different MTV thresholds, segmentation methods and imaging protocols.

c. Serious imprecision because sensitivity was based on only 145 patients with MVI, and the 95% CI corresponded to 112–177 false-negative results per 1,000 patients tested.

d. Statistical heterogeneity in specificity was not important (I²=0%).

e. Serious imprecision because specificity was based on only 191 patients without MVI, and the 95% CI corresponded to 102–176 false-positive results per 1,000 patients tested.

f. Publication bias could not be reliably assessed because fewer than 10 studies were included in each biomarker analysis; therefore, no additional rating down was applied.

**(4) Question**: Should Preoperative 18F-FDG PET/CT-derived total lesion glycolysis (TLG) be used to diagnose Histopathologically confirmed microvascular invasion in hepatocellular carcinoma in Patients with hepatocellular carcinoma undergoing liver resection or transplantation?

| Outcome | № of studies (№ of patients) | Study design | Factors that may decrease certainty of evidence | | | | | Effect per 1,000 patients tested | Test accuracy CoE |
| --- | --- | --- | --- | --- | --- | --- | --- | --- | --- |
|  |  |  | Risk of bias | Indirectness | Inconsistency | Imprecision | Publication bias | pre-test probability of 47.8% |  |
| **True positives** (patients with Histopathologically confirmed microvascular invasion in hepatocellular carcinoma) | 4 studies 245 patients | cohort & case-control type studies | serious^a^ | not serious | serious^b^ | serious^c^ | none^e^ | 287 (201 to 363) | ⨁◯◯◯ Very low^a,b,c^ |
| **False negatives** (patients incorrectly classified as not having Histopathologically confirmed microvascular invasion in hepatocellular carcinoma) |  |  |  |  |  |  |  | 191 (115 to 277) |  |
| **True negatives** (patients without Histopathologically confirmed microvascular invasion in hepatocellular carcinoma) | 4 studies 268 patients | cohort & case-control type studies | serious^a^ | not serious | serious^b^ | serious^d^ | none^e^ | 438 (397 to 470) | ⨁◯◯◯ Very low^a,b,d^ |
| **False positives** (patients incorrectly classified as having Histopathologically confirmed microvascular invasion in hepatocellular carcinoma) |  |  |  |  |  |  |  | 84 (52 to 125) |  |

#### Explanations

a. Serious risk of bias because one contributing study was at high risk of bias in patient selection and the remaining studies had incomplete reporting of patient selection, index-test threshold specification, reference-standard blinding, or flow and timing.

b. Substantial heterogeneity was observed in both sensitivity and specificity. The threshold-effect analysis also suggested that differences in study-specific TLG thresholds may have contributed to the observed heterogeneity.

c. Serious imprecision because the sensitivity estimate had a wide 95% CI of 0.42–0.76, corresponding to 115–277 false-negative results per 1,000 patients tested.

d. Serious imprecision because specificity was based on 268 patients without MVI, and its 95% CI corresponded to 52–125 false-positive results per 1,000 patients tested.

e. Publication bias could not be reliably assessed because fewer than 10 studies were included in each biomarker analysis; therefore, no additional rating down was applied.

**(5) Question**: Should Preoperative 18F-FDG PET/CT-derived **tumor-to-liver uptake ratio (TLR)** be used to diagnose Histopathologically confirmed microvascular invasion in hepatocellular carcinoma in Patients with hepatocellular carcinoma undergoing liver resection or transplantation?

| Outcome | № of studies (№ of patients) | Study design | Factors that may decrease certainty of evidence | | | | | Effect per 1,000 patients tested | Test accuracy CoE |
| --- | --- | --- | --- | --- | --- | --- | --- | --- | --- |
|  |  |  | Risk of bias | Indirectness | Inconsistency | Imprecision | Publication bias | pre-test probability of 54.1% |  |
| **True positives** (patients with Histopathologically confirmed microvascular invasion in hepatocellular carcinoma) | 4 studies 220 patients | cohort & case-control type studies | serious^a^ | not serious | serious^b^ | serious^c^ | none^f^ | 373 (292 to 438) | ⨁◯◯◯ Very low^a,b,c^ |
| **False negatives** (patients incorrectly classified as not having Histopathologically confirmed microvascular invasion in hepatocellular carcinoma) |  |  |  |  |  |  |  | 168 (103 to 249) |  |
| **True negatives** (patients without Histopathologically confirmed microvascular invasion in hepatocellular carcinoma) | 4 studies 187 patients | cohort & case-control type studies | serious^a^ | not serious | not serious^d^ | serious^e^ | none^f^ | 372 (330 to 399) | ⨁⨁◯◯ Low^a,d,e^ |
| **False positives** (patients incorrectly classified as having Histopathologically confirmed microvascular invasion in hepatocellular carcinoma) |  |  |  |  |  |  |  | 87 (60 to 129) |  |

#### Explanations

a. Serious risk of bias because one study was at high risk in patient selection, two studies were at high risk in the index-test domain, and the blinding of the histopathological reference-standard assessment was insufficiently reported across the contributing studies.

b. Serious inconsistency because sensitivity showed substantial heterogeneity across studies, with considerable variation in the definitions and thresholds of tumor-to-liver uptake ratios.

c. Serious imprecision because the sensitivity estimate was based on 220 patients with MVI, and its 95% CI corresponded to 103–249 false-negative results per 1,000 patients tested.

d. Heterogeneity in specificity was moderate and was not considered sufficiently important to warrant rating down.

e. Serious imprecision because the specificity estimate was based on 187 patients without MVI, and its 95% CI corresponded to 60–129 false-positive results per 1,000 patients tested.

f. Publication bias could not be reliably assessed because fewer than 10 studies were included in each biomarker analysis; therefore, no additional rating down was applied.

**(6) Question**: Should Preoperative imaging-measured maximum **tumor size** be used to diagnose Histopathologically confirmed microvascular invasion in hepatocellular carcinoma in Patients with hepatocellular carcinoma undergoing liver resection or transplantation?

| Outcome | № of studies (№ of patients) | Study design | Factors that may decrease certainty of evidence | | | | | Effect per 1,000 patients tested | Test accuracy CoE |
| --- | --- | --- | --- | --- | --- | --- | --- | --- | --- |
|  |  |  | Risk of bias | Indirectness | Inconsistency | Imprecision | Publication bias | pre-test probability of 51.9% |  |
| **True positives** (patients with Histopathologically confirmed microvascular invasion in hepatocellular carcinoma) | 3 studies 109 patients | cohort & case-control type studies | serious^a^ | not serious | serious^b^ | serious^c^ | none^f^ | 291 (239 to 337) | ⨁◯◯◯ Very low^a,b,c^ |
| **False negatives** (patients incorrectly classified as not having Histopathologically confirmed microvascular invasion in hepatocellular carcinoma) |  |  |  |  |  |  |  | 228 (182 to 280) |  |
| **True negatives** (patients without Histopathologically confirmed microvascular invasion in hepatocellular carcinoma) | 3 studies 101 patients | cohort & case-control type studies | serious^a^ | not serious | not serious | serious^d^ | none^f^ | 399 (356 to 433) | ⨁⨁◯◯ Low^a,d^ |
| **False positives** (patients incorrectly classified as having Histopathologically confirmed microvascular invasion in hepatocellular carcinoma) |  |  |  |  |  |  |  | 82 (48 to 125) |  |

#### Explanations

a. Serious risk of bias because two of the three studies were at high risk in the index-test domain, mainly owing to concerns regarding data-driven or non-prespecified tumor-size thresholds, and all three studies had unclear reporting of reference-standard blinding.

b. Serious inconsistency because sensitivity showed substantial heterogeneity (I²=76.0%), probably related to different tumor-size thresholds and patient characteristics.

c. Serious imprecision because sensitivity was based on only 109 patients with MVI, and its 95% CI corresponded to 182–280 false-negative results per 1,000 patients tested.

d. Serious imprecision because specificity was based on only 101 patients without MVI, and its 95% CI corresponded to 48–125 false-positive results per 1,000 patients tested.

f. Publication bias could not be reliably assessed because fewer than 10 studies were included in each biomarker analysis; therefore, no additional rating down was applied.

**(7) Question**: Should Preoperative **qualitative or comprehensive 18F-FDG PET-based assessment** be used to diagnose Histopathologically confirmed microvascular invasion in hepatocellular carcinoma in Patients with hepatocellular carcinoma undergoing liver resection or transplantation?

| Outcome | № of studies (№ of patients) | Study design | Factors that may decrease certainty of evidence | | | | | Effect per 1,000 patients tested | Test accuracy CoE |
| --- | --- | --- | --- | --- | --- | --- | --- | --- | --- |
|  |  |  | Risk of bias | Indirectness | Inconsistency | Imprecision | Publication bias | pre-test probability of 56% |  |
| **True positives** (patients with Histopathologically confirmed microvascular invasion in hepatocellular carcinoma) | 2 studies 75 patients | cohort & case-control type studies | very serious^a^ | serious^b^ | serious^c^ | serious^d^ | none^g^ | 420 (353 to 470) | ⨁◯◯◯ Very low^a,b,c,d^ |
| **False negatives** (patients incorrectly classified as not having Histopathologically confirmed microvascular invasion in hepatocellular carcinoma) |  |  |  |  |  |  |  | 140 (90 to 207) |  |
| **True negatives** (patients without Histopathologically confirmed microvascular invasion in hepatocellular carcinoma) | 2 studies 59 patients | cohort & case-control type studies | very serious^a^ | serious^b^ | not serious^e^ | serious^f^ | none^g^ | 282 (224 to 334) | ⨁◯◯◯ Very low^a,b,e,f^ |
| **False positives** (patients incorrectly classified as having Histopathologically confirmed microvascular invasion in hepatocellular carcinoma) |  |  |  |  |  |  |  | 158 (106 to 216) |  |

#### Explanations

a. Very serious risk of bias because both contributing studies were at high risk in patient selection, and additional concerns were identified regarding the conduct or interpretation of the index test. Given that all available evidence was derived from studies with important methodological limitations, the risk of bias was rated down by two levels.

b. Serious indirectness because the two studies used different PET assessment strategies, including dual-tracer PET and heterogeneous definitions of PET positivity, which were not fully consistent with a standardized preoperative 18F-FDG PET/CT index test.

c. Serious inconsistency because sensitivity differed substantially between the two studies (I²=89.3%).

d. Serious imprecision because sensitivity was based on only 75 patients with MVI, and its 95% CI corresponded to 90–207 false-negative results per 1,000 patients tested.

e. Statistical heterogeneity in specificity was not important (I²=0%), and no rating down was applied.

f. Serious imprecision because specificity was based on only 59 patients without MVI, and its 95% CI corresponded to 106–216 false-positive results per 1,000 patients tested.

g. Publication bias could not be reliably assessed because fewer than 10 studies were included in each biomarker analysis; therefore, no additional rating down was applied.

**Question**: Should Preoperative 18F-FDG PET/CT radiomics-based Rad-score be used to diagnose Histopathologically confirmed microvascular invasion in hepatocellular carcinoma in Patients with hepatocellular carcinoma undergoing liver resection or transplantation?

| Outcome | № of studies (№ of patients) | Study design | Factors that may decrease certainty of evidence | | | | | Effect per 1,000 patients tested | Test accuracy CoE |
| --- | --- | --- | --- | --- | --- | --- | --- | --- | --- |
|  |  |  | Risk of bias | Indirectness | Inconsistency | Imprecision | Publication bias | pre-test probability of 54.8% |  |
| **True positives** (patients with Histopathologically confirmed microvascular invasion in hepatocellular carcinoma) | 2 studies 125 patients | cohort & case-control type studies | serious^a^ | not serious^b^ | not serious^c^ | serious^d^ | none^f^ | 422 (373 to 460) | ⨁⨁◯◯ Low^a,b,c,d^ |
| **False negatives** (patients incorrectly classified as not having Histopathologically confirmed microvascular invasion in hepatocellular carcinoma) |  |  |  |  |  |  |  | 126 (88 to 175) |  |
| **True negatives** (patients without Histopathologically confirmed microvascular invasion in hepatocellular carcinoma) | 2 studies 103 patients | cohort & case-control type studies | serious^a^ | not serious^b^ | not serious^c^ | serious^e^ | none^f^ | 344 (298 to 380) | ⨁⨁◯◯ Low^a,b,c,e^ |
| **False positives** (patients incorrectly classified as having Histopathologically confirmed microvascular invasion in hepatocellular carcinoma) |  |  |  |  |  |  |  | 108 (72 to 154) |  |

#### Explanations

a. Serious risk of bias because both studies had unclear risk regarding patient selection, blinding of the histopathological reference-standard assessment, and flow and timing. In addition, the validation-cohort denominators were not completely reported, and the diagnostic 2×2 data were reconstructed from the available sample sizes, sensitivities and specificities.

b. The populations, radiomics-based index tests, target condition and histopathological reference standards were generally consistent with the review question. Although the two studies used different radiomics signatures and model-defined thresholds, both evaluated PET/CT-based Rad-scores for the preoperative prediction of MVI.

c. Statistical inconsistency was not considered serious because the pooled sensitivity and specificity estimates showed no important statistical heterogeneity (I²=0%). However, this finding should be interpreted cautiously because only two studies were available.

d. Serious imprecision because sensitivity was based on only 125 patients with MVI, and its 95% CI corresponded to 88–175 false-negative results per 1,000 patients tested.

e. Serious imprecision because specificity was based on only 103 patients without MVI, and its 95% CI corresponded to 72–154 false-positive results per 1,000 patients tested.

f. Publication bias could not be reliably assessed because fewer than 10 studies were included in each biomarker analysis; therefore, no additional rating down was applied.
